# Interleukin-10 enhances recruitment of immune cells in the neonatal mouse model of obstructive nephropathy

**DOI:** 10.1038/s41598-024-55469-9

**Published:** 2024-03-06

**Authors:** Maja Wyczanska, Franziska Thalmeier, Ursula Keller, Richard Klaus, Hamsa Narasimhan, Xingqi Ji, Barbara U. Schraml, Lou M. Wackerbarth, Bärbel Lange-Sperandio

**Affiliations:** 1 Department of Pediatrics, Dr. v. Hauner Children’s Hospital, University Hospital, LMU Munich, Lindwurmstraße 4, 80337 Munich, Germany; 2 Biomedical Center, Institute for Cardiovascular Physiology and Pathophysiology, Faculty of Medicine, LMU Munich, 82152 Planegg-Martinsried, Germany

**Keywords:** Cytokines, Inflammation, Nephrology, Kidney

## Abstract

Urinary tract obstruction during renal development leads to inflammation, leukocyte infiltration, tubular cell death, and interstitial fibrosis. Interleukin-10 (IL-10) is an anti-inflammatory cytokine, produced mainly by monocytes/macrophages and regulatory T-cells. IL-10 inhibits innate and adaptive immune responses. IL-10 has a protective role in the adult model of obstructive uropathy. However, its role in neonatal obstructive uropathy is still unclear which led us to study the role of IL-10 in neonatal mice with unilateral ureteral obstruction (UUO). UUO serves as a model for congenital obstructive nephropathies, a leading cause of kidney failure in children. Newborn *Il-10*^*−/−*^ and C57BL/6 wildtype-mice (WT) were subjected to complete UUO or sham-operation on the 2nd day of life. Neonatal kidneys were harvested at day 3, 7, and 14 of life and analyzed for different leukocyte subpopulations by FACS, for cytokines and chemokines by Luminex assay and ELISA, and for inflammation, programmed cell death, and fibrosis by immunohistochemistry and western blot. Compared to WT mice, *Il-10*^*−/−*^ mice showed reduced infiltration of neutrophils, CD11b^hi^ cells, conventional type 1 dendritic cells, and T-cells following UUO. *Il-10*^*−/−*^ mice with UUO also showed a reduction in pro-inflammatory cytokine and chemokine release compared to WT with UUO, mainly of IP-10, IL-1α, MIP-2α and IL-17A. In addition, *Il-10*^*−/−*^ mice showed less necroptosis after UUO while the rate of apoptosis was not different. Finally, α-SMA and collagen abundance as readout for fibrosis were similar in *Il-10*^*−/−*^ and WT with UUO. Surprisingly and in contrast to adult *Il-10*^*−/−*^ mice undergoing UUO, neonatal *Il-10*^*−/−*^ mice with UUO showed a reduced inflammatory response compared to respective WT control mice with UUO. Notably, long term changes such as renal fibrosis were not different between neonatal *Il-10*^*−/−*^ and neonatal WT mice with UUO suggesting that IL-10 signaling is different in neonates and adults with UUO.

## Introduction

Congenital obstructive nephropathy is the main cause of kidney failure in infants and children, impairs fetal nephrogenesis and induces severe disruption of nephron maturation leading to nephron loss^1–5^. Unilateral ureteral obstruction (UUO) performed in neonatal mice serves as a model for congenital obstructive nephropathy. UUO induces sterile inflammation, kidney injury, cell death, and renal fibrosis, leading to loss of nephron mass in the developing kidney with obstruction^4,6,7^.

Interleukin-10 (IL-10) is an immunosuppressive cytokine, initially described as a T helper 2 derived cytokine and now known to be produced by various cell types^8,9^. Major sources of IL-10 include T helper cells, monocytes, macrophages, and dendritic cells. IL-10 limits innate as well as adaptive immune responses and protects the host from immune-related tissue damage^10,11^. IL-10 dampens immune responses mainly through blocking activation of inflammatory pathways^12,13^. IL-10 is mostly known for its anti-inflammatory functions, but under certain circumstances it exhibits pro-inflammatory functions as well^14–16^. IL-10 has the capacity to elicit pro-inflammatory effects, including stimulation of granzyme B and interferon-γ production by CD8^+^ T cells^17^. Additionally, high-dose IL-10 treatment in patients with inflammatory disorders can be associated with undesired pro-inflammatory effects^16,18^.

IL-10 has been shown to play an important part in several renal diseases, one example being renal ischemia–reperfusion injury^19,20^. An association between IL-10 polymorphisms and the risk of developing diabetic nephropathy has been shown^21^. In human fetuses with urethral valves, a severe form of lower urinary tract obstruction, increased urinary levels of IL-10 were measured^22^.

Recently it has been shown that IL-10 deficiency leads to an increase of macrophage and T-cell infiltration into kidneys following UUO in adult mice, thereby increasing the inflammatory response^23^. Thus, IL-10 seems to reduce immune responses and leukocyte infiltration into the kidney. IL-10 suppresses inflammatory processes by inhibiting the secretion of a broad variety of pro-inflammatory chemokines and cytokines, like interperitoneally-10 (IP-10/CXCL10), macrophage inflammatory protein (MIP-2α/CXCL2), and IL-1α^24,25^. Additionally, cytokines released by macrophages, like IL-6 and IL-18 are also inhibited by IL-10^26,27^. Vice versa the knock-out of IL-10 leads to an increased expression of T cell produced cytokines IL-17 and IL-22^28^.

A central player in obstructive nephropathy is tumor necrosis factor-α (TNF-α), which mediates the inflammatory response by promoting the activation and recruitment of immune cells^29^. The balance of TNF-α and IL-10 is important for the maintenance of immune homeostasis^30^. To counteract exuberant production of TNF-α following injury, IL-10 is able to suppress TNF-α secretion through different mechanisms^31^.

One of the hallmarks of UUO is cell death in various forms. Apoptosis is increased in the neonatal obstructed kidney^4^. For IL-10, a blocking effect in inducing apoptosis has been reported in several disorders^32–34^. Most importantly, in the adult model of UUO, IL-10 has been shown to attenuate apoptosis through regulating endoplasmic reticulum (ER) stress as demonstrated by the upregulation of ER markers including 78-kDa glucose-regulated protein (GRP78)^23^. Besides apoptosis there are other forms of regulated cell death that play a role in neonatal UUO^4^. Necroptosis, a necrotic form of cell death, is mediated by the receptor interacting serine/threonine-protein-kinase-3 (RIPK3) and unlike apoptosis triggers inflammation^35^. Until now, IL-10 has not been investigated in relation to necroptosis, but it has been shown that IL-10 can prevent necrosis in murine experimental acute pancreatitis implying a potential protective role of IL-10 in necroptosis^36^.

Finally, IL-10 has been shown to inhibit organ fibrosis in several animal models including UUO in adult mice^37–40^. In UUO, activation of fibroblasts and secretion of additional extracellular matrix components induce development of renal tubulointerstitial fibrosis^41^, a process which is attenuated in the presence of IL-10^23,42^.

Because of striking differences in the pathogenesis of UUO in adults and neonates, we set out to investigate the role of IL-10 in the neonatal mouse model of congenital obstructive nephropathy. Our results surprisingly reveal a pro-inflammatory role of IL-10 inducing immune cell recruitment into the obstructed kidney in neonatal mice with UUO without altering the course of renal fibrosis development following UUO.

## Materials and methods

### Experimental protocol

*Il-10*^*−/−*^ mice and WT mice (C57BL/6J) were subjected to complete left ureteral obstruction or sham operation under general anesthesia with isoflurane (3–5% v/v) and oxygen (0.8 L/min) on the second day of life, as described before^43^. The animals received carprofen (5 mg/kg) to alleviate possible pain after the surgery. The sex distribution was equal in both groups. All mice were raised in the same environmental condition, group-housed in the same room, under the same controlled temperature (20–22 °C) and photoperiods (12:12-h light–dark cycle) and fed with the same chow and water. After recovery, neonatal mice were returned to their mothers until sacrifice on day 3, 7 and 14 of life. The animals were sacrificed by cervical dislocation. The weight of the kidneys harvested was on average between 15 mg (d3) and 65 mg (d14). *Il-10*^*−/−*^ mice with a C57BL/6 background (B6.129P2-Il10tm1Cgn/J) were obtained from Charles River Laboratories (Sulzfeld, Germany). All experiments were performed according to national animal protection laws and the guidelines of animal experimentation established and approved by governmental committee (Regierungspräsidium von Oberbayern) (Az ROB-55.2-2532.Vet_02-19-109). This study is reported in accordance with ARRIVE guidelines.

### IL-10 ELISA assay

WT mice were either subjected to UUO or not operated. At day 7 mice were sacrificed by decapitation to collect blood samples into serum-separating tubes; additionally, blood samples from adult mice were collected (neonatal UUO n = 4, neonatal mice non-operated n = 5, adult mice n = 3). The tubes were inverted 5 times, left standing for 30 min and centrifugated at 8000*g* for 90 s. The serum was collected and used for an IL-10 ELISA assay (R&D Systems M1000B-1, Minneapolis, MN) as per manufacturer’s instructions.

### Cell isolation

Kidneys were isolated from neonatal *Il-10*^*−/−*^ and WT mice without perfusion at day 3, 7, and 14 (n = 3 for each group) and cut into small pieces. The samples were processed as described previously^44^. In brief, kidneys were digested in 2 ml of RPMI (Thermo Fisher Scientific, MA) with 200 U/ml collagenase IV (Worthington Biochemical, NJ) and 0.2 mg/ml DNAse I (Roche, Switzerland) for 1 h at 37 °C while shaking (120 rpm). After digestion, cells were passed through a 70-mm strainer and washed once with FACS buffer. Leukocytes were enriched using a 70%–37%–30% Percoll gradient by centrifugation (2000 rpm for 30 min at room temperature). Cells were collected at the 70%–37% interface. Percoll (100%) was prepared by adding nine parts of Percoll (GE Healthcare, IL) to one part of 10× concentrated PBS. After Percoll enrichment, cells were washed once and resuspended in FACS buffer (PBS with 1% FBS, 2.5 mM EDTA (Invitrogen, CA), 0.02% sodium azide (Sigma-Aldrich, MO)) for analysis.

### Flow cytometry

For surface staining, cells were incubated with 50 µl purified anti-mouse CD16/32/FcBlock for 10 min at 4 °C, as described previously^45^. Additional antibodies were then added in FACS buffer to a final volume of 100 µl at 4 °C for 20 min. After staining, cells were washed twice and resuspended in FACS buffer for analysis. Dead cells were excluded from analysis by fixable viability dye eFluor™ 780 (Thermo Fisher Scientific, MA). Flow cytometry was performed on an LSR Fortessa (BD Biosciences, NJ) with subsequent data analysis using FlowJo software (Tree Star). Cells were quantified by using CountBright Absolute Counting Beads (Thermo Fisher Scientific, MA). The following antibodies were purchased from Biolegend: anti-CD45.2-R-phycoerythrin-cyanine 7 (PECy7) (clone: 104), anti-MHCII I-A/I-E-AF700 (clone: M5/114.15.2), anti-CD11c-BV786 (clone: N418), anti–CD3e-PECy5 (clone: 145-2C11), anti-CD19-BV650 (clone: 6D5), anti-Ly6G-Peridinin-chlorophyll (PerCP)-Cy5.5 (clone: 1A8), anti-F4/80-AF647 (clone: BM8), anti-CD24-BUV395 (clone: M1/69), anti-CD64-PE (clone: X54-5/7.1), anti-Ly6C-BV605 (clone: HK1.5). The following antibodies were purchased from BD Biosciences: anti-CD11b-Brilliant UltraViolet (BUV) 737 (clone: M1/70).

### Identification of infiltrating macrophages and T-lymphocytes

The abundance of infiltrating macrophages and T-lymphocytes in the neonatal kidney was examined by immunohistochemical staining. Formalin-fixed, paraffin-embedded kidney sections were subjected to antigen retrieval and incubated with either rat anti-mouse F4/80 antibody (Cell Signaling Technology #70076, MA, 1:200) or anti-human CD3 antibody (Serotec MCA 1477, Bio-Rad, UK) (n = 10 for each group). Specificity was assessed through simultaneous staining of control sections with an unspecific, species-controlled primary antibody. Biotinylated mouse anti-rabbit IgG (Santa Cruz Biotechnology sc2491, TX) and goat anti-rat IgG (Southern Biotech 3050-8, AL) were used as secondary antibodies. Sections were incubated with ABC reagent (Vectastain PK6100, Vector Laboratories, CA), detected with DAB (CD3: Dako, Agilent Technologies, CA, #K3468) (F4/80: Vectastain, Vector Laboratories, CA) and counterstained with methylene blue or hematoxylin. Images were taken using the LEICA DM1000 microscope and the digital camera (LEICA ICC50HD, Germany). Macrophages and CD3-positive lymphocytes in cortex and medulla were counted in twenty nonoverlapping high-power fields at 400× magnification and were analyzed in a blinded manner. Data were expressed as the mean score ± SEM per 20 high-power fields.

### Cytokine and chemokine protein expression

Kidneys of UUO and control mice were harvested on 3, 7 and 14 days of life (n = 3 in each group) as described previously^4^. In brief, kidneys were homogenized in protein lysis buffer (Tris 50 mM, Na_2_VO_2_ 1 mM, 2% SDS) containing protease inhibitor cocktail (Roche, Switzerland, #1836153). The protein content of the supernatants was measured using the BCA Protein Assay Kit (ThermoFisher, MA, Pierce #23225). The supernatants were diluted to contain the same protein concentration. Expression of 36 cytokines and chemokines (i.a. IP-10, MIP-2α, IL-1α, IL-17A, IL-4, eotaxin, ENA-78) was determined by Luminex multiplex assay (ThermoFisher, MA, cat. No. EPX360-26092-901) as per manufacturer’s instructions.

### Detection of apoptosis

Apoptotic cells were detected by the terminal deoxynucleotidyl transferase (TdT)-mediated dUTP-biotin nick end labeling (TUNEL) assay, as described before^4^. Briefly, 4% formalin-fixed tissue sections were deparaffinized and rehydrated in ethanol, followed by incubation with proteinase K. After quenching, equilibration buffer and working strength enzyme (ApopTag Peroxidase In Situ Apoptosis Detection Kit, Millipore, MA) were applied. If the nuclei were stained black and displayed typical apoptotic morphology cells were regarded as TUNEL-positive. Apoptosis in each kidney was calculated by counting the number of TUNEL-positive tubular and interstitial cells in 20 sequentially selected fields at 400× magnification and expressed as the mean number ± SEM per 20 high-power fields using the LEICA DM1000 microscope and the digital camera (LEICA ICC50HD, Germany) (n = 10 for each group).

### Measurement of interstitial fibrosis

Interstitial collagen deposition was measured in Masson’s trichrome-stained sections as described before^6^. Digital images of the sections were superimposed on a grid, and the number of grid points overlapping interstitial blue-staining collagen was recorded for each field. In addition, formalin-fixed and paraffin embedded sections were subjected to antigen retrieval and incubated with mouse anti-mouse α-smooth muscle actin antibody (Sigma Aldrich, Germany, A2547, 1:400) as shown before^6^. Biotinylated horse anti-mouse IgG (Santa Cruz, Germany) was used as a secondary antibody. Sections were incubated with ABC reagent (Vectastain PK 6100, Vector Laboratories, CA), detected with AEC-Mix Romulin (Biocare 901-RAEC810-082117, CA) and counterstained with hematoxylin. Digital images of the sections (n = 10 in each group) were superimposed on a grid, and the number of grid points overlapping collagen I fibers or α-smooth muscle actin fibers was recorded for each field. Twenty non-overlapping high-power fields at 400× magnification were analyzed in a blinded fashion. Data were expressed as the mean score ± SEM per 20 high power fields.

### Western immunoblotting

*Il-10*^*−/−*^ and WT male and female neonatal mice underwent UUO surgery or sham operation at the second day of life for Western blot analysis. Kidneys were harvested on 3, 7 and 14 days of life (n = 3 in each group), homogenized in protein lysis buffer (Tris 50 mM, Na_2_VO_2_ 1 mM, 2% SDS) containing protease inhibitor cocktail (Roche, Switzerland, #1836153). The protein content of the supernatants was measured using the BCA Protein Assay Kit (ThermoFisher, MA, Pierce #23225). 20 μg of protein were separated on polyacrylamide gels at 160 V for 45 min and blotted onto nitrocellulose membranes (100 mA per gel, 120 min). After blocking antibody-specific for 2 h in Tris-buffered saline with Tween-20 containing 5% nonfat dry milk and/or BSA, blots were incubated with primary antibodies 2 h at room temperature or at 4 °C overnight. PARP antibody (Cell Signaling Technology #9542, MA, 1:500), RIPK3/RIP3 antibody (Novus Biologicals #77299, Germany, 1:2000), GRP78/BiP antibody (Cell Signaling Technology #3183, MA, 1:1000), α-SMA antibody (Sigma Aldrich A2547, Germany, 1:5000), β-catenin antibody (Upstate Biotechnology 05-665, Fisher Specific, NY, 1:200), and TGF-β antibody (Cell Signaling Technology #3711, MA, 1:2000) were used for western blot analysis. GAPDH (Biodesign Meridian LifeScience H86540M, Memphis, TN, 1:40,000) was used as an internal loading control and to normalize samples. Blots were washed with Tris-buffered saline with Tween-20 and incubated with horseradish peroxidase-conjugated secondary antibody for 1 h at room temperature. Immune complexes were detected using enhanced chemiluminescence method. Blots were exposed to x-ray films (Kodak, Germany), the films were scanned, and protein bands were quantified using the densitometry program Image J. Each band represents one single neonatal mouse kidney.

### Statistical analysis

Data are presented as x-fold increase after UUO. For this the results obtained from analysis of UUO kidneys are divided by the average of sham results. This form of presentation allows us to show the actual impact of UUO with the sham-measurements as basis. Data are presented as mean ± standard error. Comparisons between groups were made using one-way analysis of variance followed by the Student–Newman–Keuls test. Comparisons between left and right kidneys were performed using the Students *t*-test for paired data. Statistical significance was defined as *p* < 0.05.

## Results

### Neonatal UUO induces upregulation of IL-10 serum concentrations in neonatal WT mice

**Figure 1:**
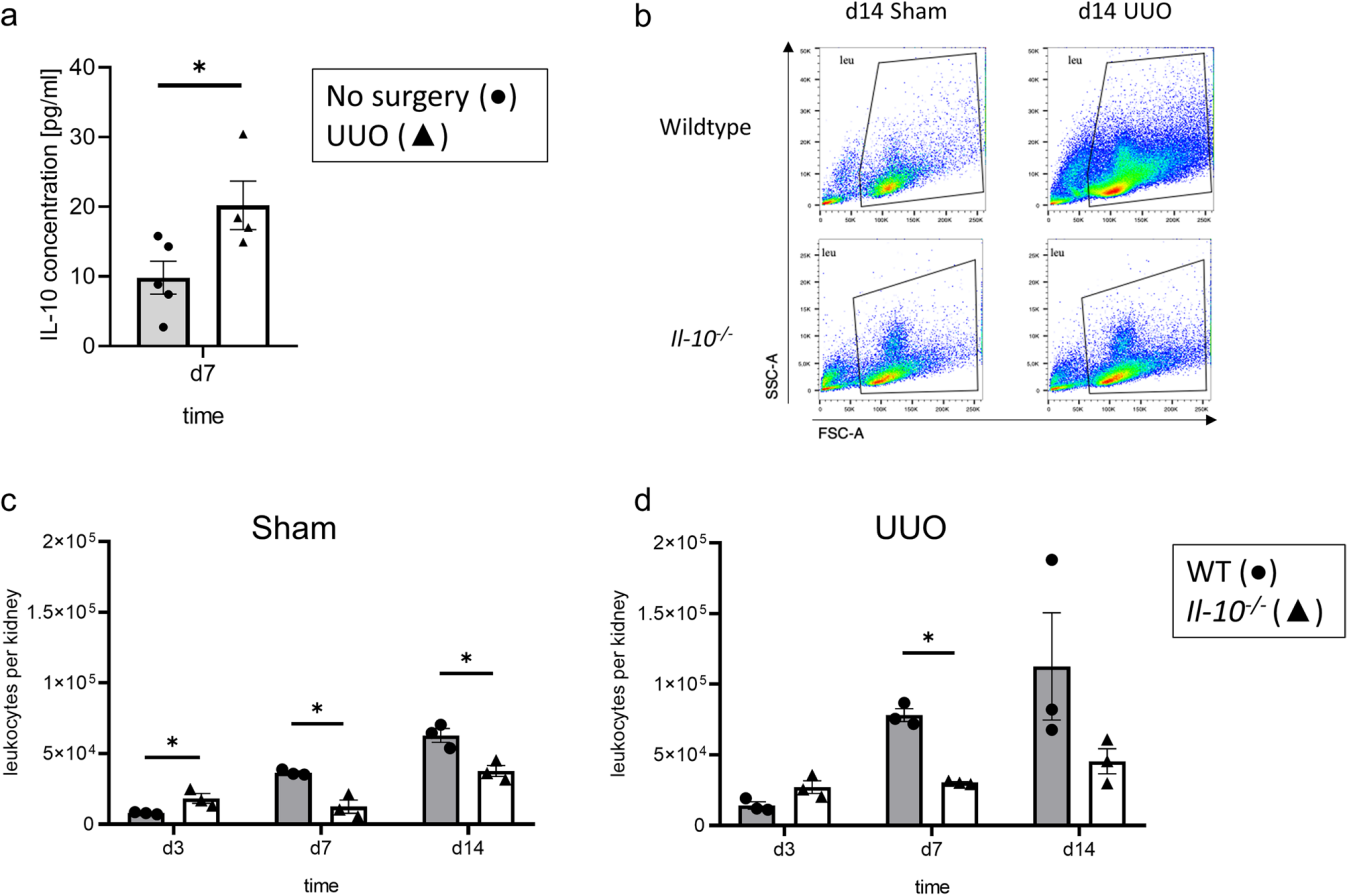
Reduced numbers of leukocytes in *Il-10*^*−/−*^ mice with unilateral ureteral obstruction. Neonatal WT mice were subjected to UUO on the 2nd day of life and blood serum samples for an ELISA assay were collected at d7 of life. Serum samples of non-operated mice were used as control. IL-10 concentration was significantly higher in UUO samples compared to non-operated controls (**a**). n = 4 for UUO; n = 5 for non-operated controls. Whole kidneys were harvested for the FACS analysis on day 3, 7, and 14. Cells were gated on live CD45.2^+^ cells for WT and *Il-10*^*−/−*^ sham-operated and UUO kidneys on d14 (**b**). Leukocyte count was lower in the *Il-10*^*−/−*^ sham-operated kidneys at all time points (**c**) and for the UUO kidneys on d7 (**d**) in comparison to WT. n = 3; *p < 0.05. Data are presented as individual points with mean ± SEM.

We performed unilateral ureteral obstruction (UUO) in neonatal mice at day 2 of life and measured concentrations of serum IL-10 at day 7 of life by ELISA. IL-10 concentrations in UUO mice increased significantly in comparison to neonatal non-operated control mice (Fig. 1a) confirming earlier reports in adult mice with UUO^23^. We also measured IL-10 concentrations in serum of adult non-operated mice; notably, the IL-10 concentrations were too low to detect compared to IL-10 concentrations in neonatal mice.

### Leukocyte infiltration into obstructed kidneys of neonatal mice is reduced in the absence of IL-10

**Figure 2:**
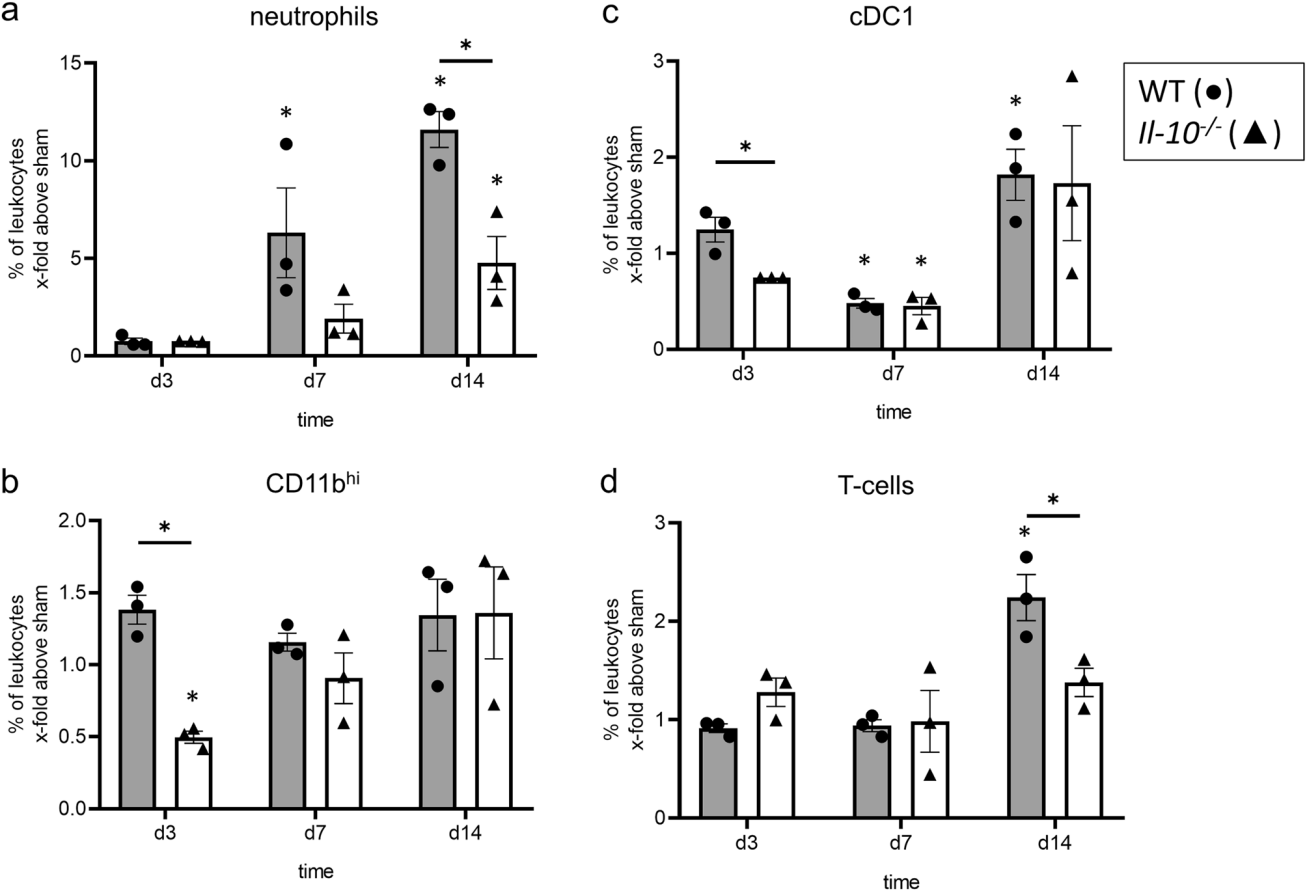
IL-10 induces the infiltration of neutrophiles, macrophages, dendritic cells and T-cells after neonatal UUO. Neonatal mice were subjected to UUO or sham operation. Frequency of renal Ly6G^+^ neutrophils (**a**), CD11b^hi^ cells (**b**), cDC1 dendritic cells (**c**), and CD3^+^ T-cells (**d**) at the indicated ages are shown. Neutrophils infiltrated the obstructed kidney; the infiltration was lower in the *Il-10*^*−/−*^ than in the WT (**a**). UUO induced infiltration of CD11b^hi^ cells (**b**) and dendritic cells (**c**); the infiltration was lower in the *Il-10*^*−/−*^ compared to the WT on d3. T-cells infiltrated the kidney following UUO; T-cell infiltration was lower in the *Il-10*^*−/−*^ in comparison to WT on d14 (**d**). Results are indicated as x-fold increase above sham operated control; n = 3; *p < 0.05. Data are presented as individual points with mean ± SEM. Standalone * represents significant differences between Sham and UUO results.

**Figure 3:**
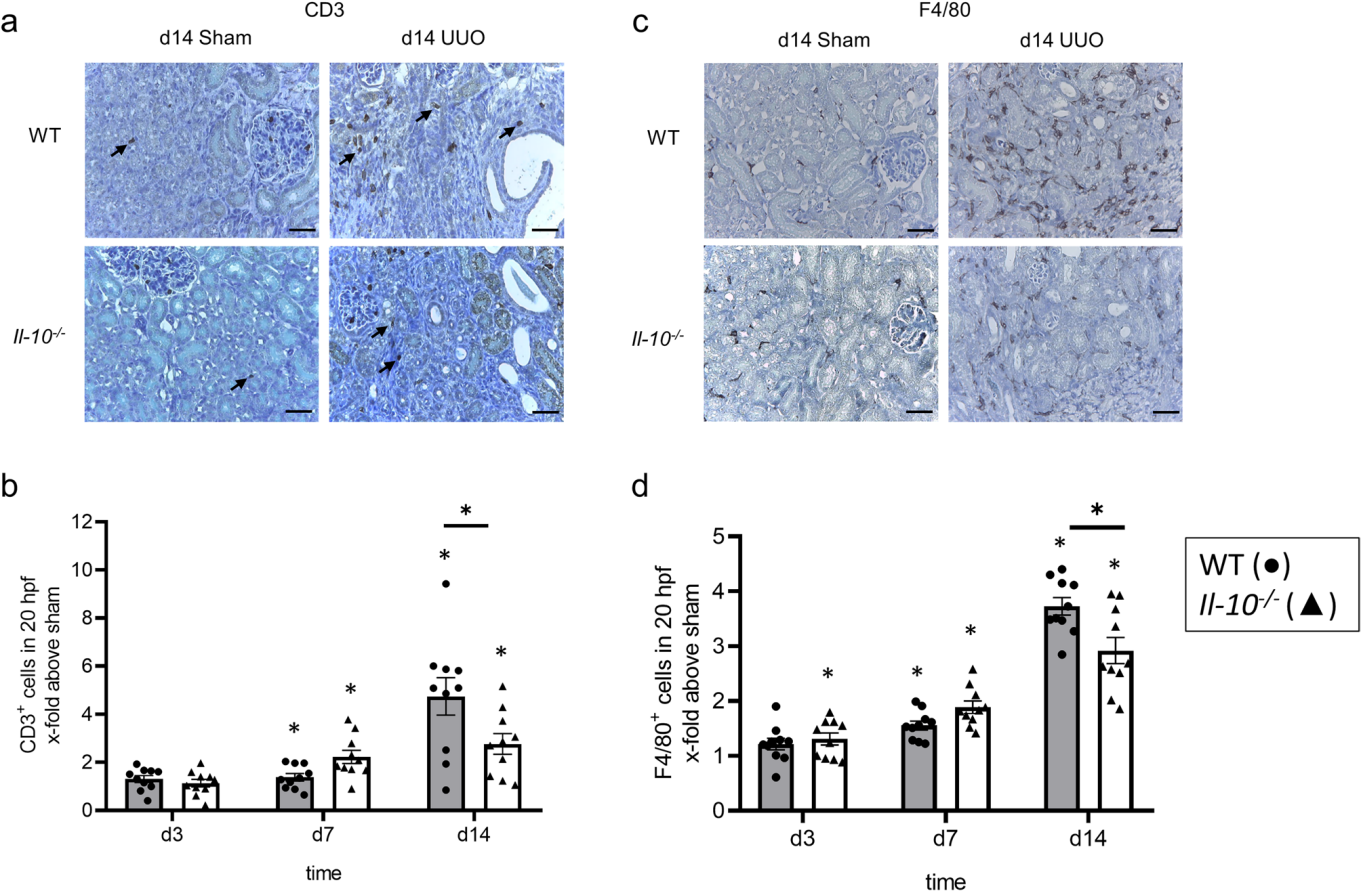
Immune cell infiltration of CD3^+^ and F4/80^+^ cells. Immunohistological staining for CD3 (positive cells marked with arrows), a marker for T-cells and F4/80, a macrophage and dendritic cells marker, of WT sham and UUO, and *Il-10*^*−/−*^ sham and UUO mice on d14 (**a**,**c**). Neonatal UUO induced infiltration of CD3^+^ T-cells in the kidney; the infiltration was lower for *Il-10*^*−/−*^ compared to WT on d14 (**b**). Following UUO F4/80^+^ cells infiltrated the kidney, with more cells infiltrating *Il-10*^*−/−*^ on d7 and fewer cells on d14 (**d**). The gating strategy for these leukocyte subpopulations can be found in Supplementary Fig. S2. Results are indicated as x-fold increase above sham operated control in 20 hpfs (×400); n = 10; *p < 0.05. Data are presented as individual points with mean ± SEM. Bar = 100 µm. Standalone * represents significant differences between Sham and UUO results.

Because IL-10 deficiency led to an increase in recruited leukocytes into kidneys from adult mice subjected to UUO^23^, we profiled leukocyte infiltration in neonatal *Il-10*^*−/−*^ and WT mouse kidneys with UUO using FACS analysis. Surprisingly, neonatal *Il-10*^*−/−*^ mice showed less leukocyte infiltration in both sham-operated and UUO-kidneys compared to respective WT mice (Fig. 1b–d). We also performed a steady state analysis; no differences were found between sham-operated and non-operated kidneys in both lines (Supplementary Material Fig. S1) indicating that there is no induction of leukocyte recruitment in sham-operated neonatal *Il-10*^*−/−*^ and WT mice. Next, we analyzed leukocyte subpopulations including Ly6G^+^ neutrophils, CD11b^hi^ cells, CD3^+^ T-cells, and type 1 and type 2 conventional dendritic cells, including CD11b^hi^CD64^+^ DC like cell type and F4/80 macrophages (here called cDCs). The gating strategy is shown in Supplementary Fig. S2, as recently reported^46^. UUO induced an increase in the infiltration of all leukocyte subtypes investigated (Fig. 2). This was true for the frequency, which is defined as the percentage of subtype number to total leukocyte number. We then studied the influence of IL-10 on the infiltration of leukocyte subsets. Compared to UUO in neonatal WT mice, infiltration of neutrophils was most prominently reduced in d14 *Il-10*^*−/−*^ mice with UUO (Fig. 2a). We also found some reduction in the infiltration of CD11b^hi^, cDCs, and T-cells in neonatal *Il-10*^*−/−*^ compared to WT mice with UUO (Fig. 2b–d). These findings on leukocyte subset infiltration were confirmed for CD3^+^ and F4/80^+^ cells by immunohistochemistry (Fig. 3), suggesting that loss of IL-10 attenuated leukocyte infiltration into obstructed kidneys of neonatal mice which is the opposite to findings in adult mice with UUO^23^. Results of these analyzes in sham-operated kidneys only are displayed in Supplementary Fig. S3.

### Release of cytokines and chemokines after UUO is reduced in neonatal Il-10^−/−^ mice

**Figure 4:**
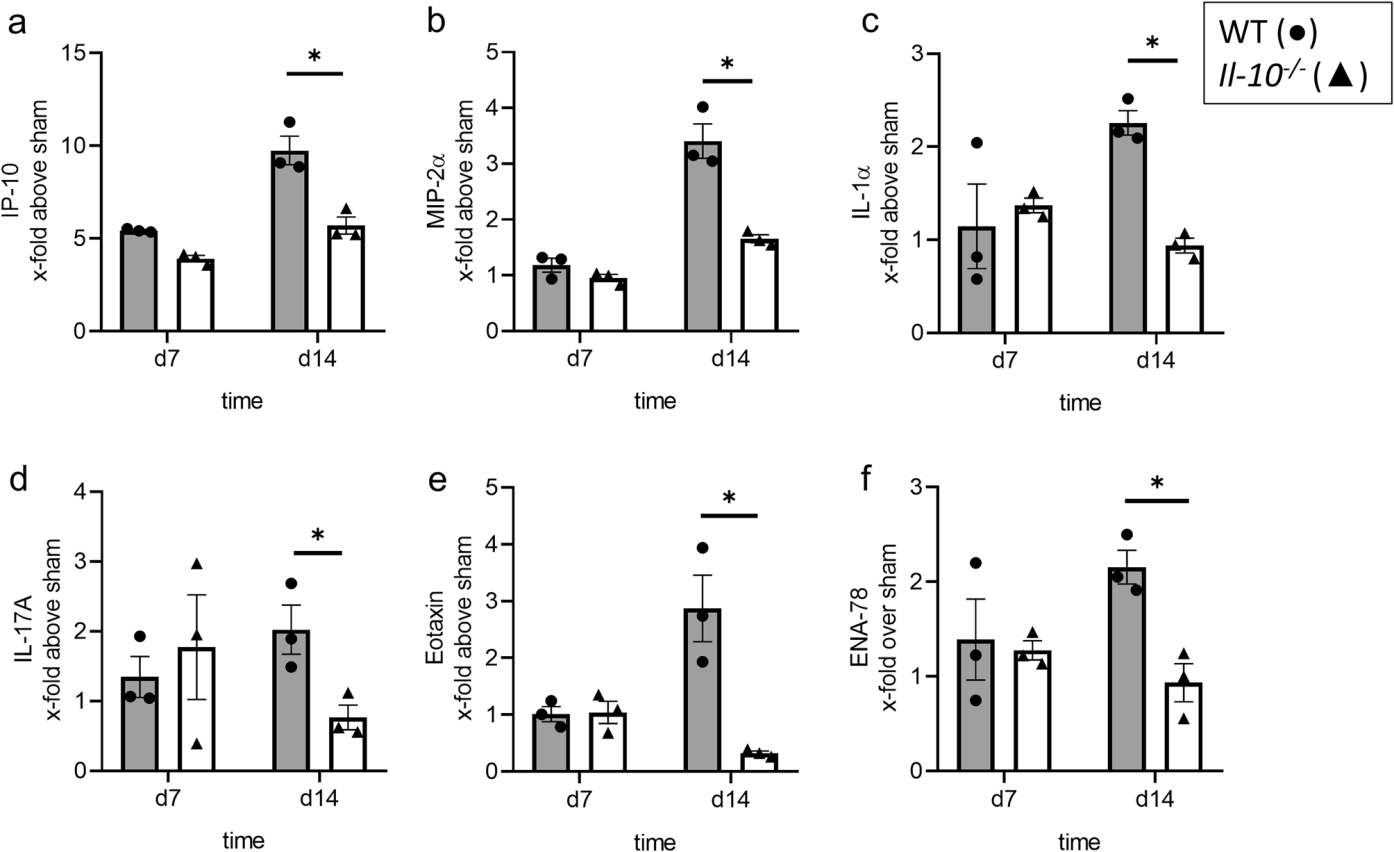
IL-10 influences cytokine and chemokine release after neonatal UUO. Whole sham-operated and UUO kidneys of neonatal mice were harvested and analyzed for cytokine and chemokine concentration. IP-10/CXCL10 concentration increased markedly after UUO, with a lower increase in *Il-10*^*−/−*^ kidneys compared to WT (**a**). UUO induced IL-1α release in WT kidneys, but not in *Il-10*^*−/−*^ on d14 (**b**). MIP-2α/CXCL2 increased following UUO, the increase was lower in the *Il-10*^*−/−*^ kidneys in comparison to WT (**c**). Neonatal UUO induced IL-17A release, the concentration of IL-17A was lower in *Il-10*^*−/−*^ compared to WT on d14 (**d**). Following UUO concentrations of the chemokines eotaxin/CCL11 (**e**) and ENA-78/CXCL5 (**f**) increased in WT mice, while no increase was observed in *Il-10*^*−/−*^ mice on d14. Concentration is indicated as x-fold increase above sham operated control; n = 3; *p < 0.05. Data are presented as individual points with mean ± SEM. Standalone * represents significant differences between Sham and UUO results.

To assess released cytokines and chemokines in the kidney after neonatal UUO in the presence and absence of IL-10, a Luminex analysis of 36 chemokines and cytokines was performed in *Il-10*^*−/−*^ and WT mice at day 7 and 14 of life (Fig. 4a–f). At day 7, UUO provoked a marked increase in IP-10 release in both *Il-10*^*−/−*^ and WT mice, while we observed no substantial release of MIP-2α, IL-1α, IL-17A, eotaxin, and ENA-78 in *Il-10*^*−/−*^ and WT mice at day 7 of life (Fig. 4a–f). All here investigated cytokines were released at much higher quantities in WT mice with UUO at day 14 of life compared to respective *Il-10*^*−/−*^ mice with UUO at day 14 of life (Fig. 4a–f), suggesting a potential pro-inflammatory role of IL-10 in obstructed kidneys of neonatal mice. Expression of these chemokines and cytokines in sham-operated kidneys only are displayed in Supplementary Fig. S4. We also measured interleukin-4 (IL-4) in obstructed kidneys (Supplementary Fig. S5), however it did show neither a response to UUO nor a difference between *Il-10*^*−/−*^ and WT kidneys.

### Necroptosis but not apoptosis is reduced in neonatal Il-10^−/−^ mice following UUO

**Figure 5:**
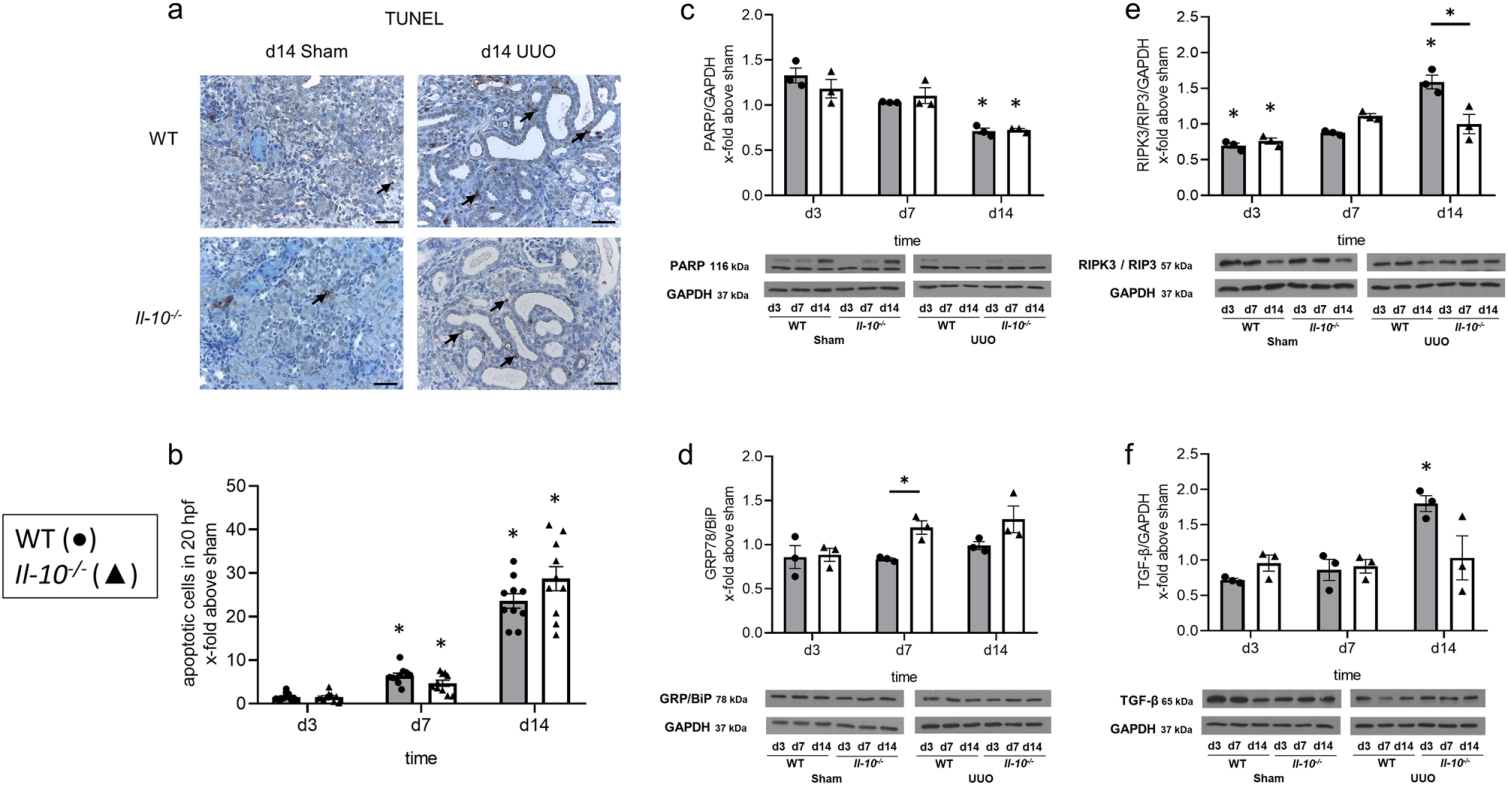
Cell death in neonatal *Il-10*^*−/−*^ mice in comparison to the WT. Apoptotic cells in sham-operated and UUO kidneys were detected by TUNEL staining in sections. TUNEL-positive cells (marked with arrow) in WT sham and UUO, and in *Il-10*^*−/−*^ sham and UUO appeared in distal tubules (**a**). Number of tubular apoptotic nuclei increased following UUO, without significant differences between neonatal *Il-10*^*−/−*^ and WT kidneys (**b**). Whole kidneys were processed for western blot analysis at day 3, 7 and 14. Expression of PARP, a marker for apoptosis, which is cleaved in the process of cell death, decreased following UUO, but was not significantly different between *Il-10*^*−/−*^ and WT mice (**c**). ER stress, measured by the expression of GRP78/BiP, increased following UUO and was increased in *Il-10*^*−/−*^ kidneys on d7 compared to WT (**d**). Expression of RIPK3, a marker for necroptosis, increased after UUO, but with a weaker increase in the *Il-10*^*−/−*^ compared to WT (**e**). UUO induced TGF-β expression with a decreasing trend for *Il-10*^*−/−*^ in comparison to WT (**f**). The shown western blot images are cropped, for uncropped western blots see Supplementary Figs. S4–S7 online. Expression is indicated as x-fold increase above sham operated control; n = 3 for western blot analysis and n = 10 for immunohistochemical staining; *p < 0.05. Data are presented as individual points with mean ± SEM. Bar = 100 µm. Standalone * represents significant differences between Sham and UUO results.

Next, we assessed the rate of apoptosis and necroptosis in neonatal mice following UUO. Tubular apoptosis in neonatal *Il-10*^*−/−*^ and WT sham-operated and UUO kidneys was measured by TUNEL staining for apoptotic nuclei (Fig. 5a,b). The number of tubular apoptotic cells in neonatal kidneys following UUO increased in a similar fashion in both *Il-10*^*−/−*^ and WT mice (Fig. 5a,b). Additionally, full length expression of PARP as a read out for apoptosis and measured by western blot (Fig. 5c) (uncropped western blot image: Supplementary Fig. S6) showed no differences between obstructed kidneys from neonatal *Il-10*^*−/−*^ and WT mice. Furthermore, ER stress, a possible source for apoptosis, was measured by western blot using the marker GRP78/BiP. UUO induced ER stress in neonatal kidneys with a small but significant increase of GRP78/BiP expression in *Il-10*^*−/−*^ compared to WT mice at d7 of life, while no difference could be found for d3 and d14 (Fig. 5d) (uncropped western blot image: Supplementary Fig. S7).

Necroptosis, another form of cell death, was measured by expression of RIPK3/RIP3 using western blot (Fig. 5e) (uncropped western blot image: Supplementary Fig. S8). We found a significant decrease in RIPK3 expression in obstructed kidneys of neonatal *Il-10*^*−/−*^ compared to WT mice only for day 14 of life but not at earlier time points. Finally, expression of TGF-β, which is involved in both apoptotic and necroptotic cell death pathways, did not show any significant differences, but a decreasing trend in obstructed kidneys of neonatal *Il-10*^*−/−*^ compared to WT mice (Fig. 5f) (uncropped western blot image: Supplementary Fig. S9). Expression of these cell death markers in sham-operated kidneys only is displayed in Supplementary Fig. S10. From these results we conclude that IL-10 has only a minor influence on cell death following UUO in neonatal mouse kidneys.

### Renal fibrosis increased after neonatal UUO with no substantial differences between Il-10^−/−^ and WT mice

**Figure 6:**
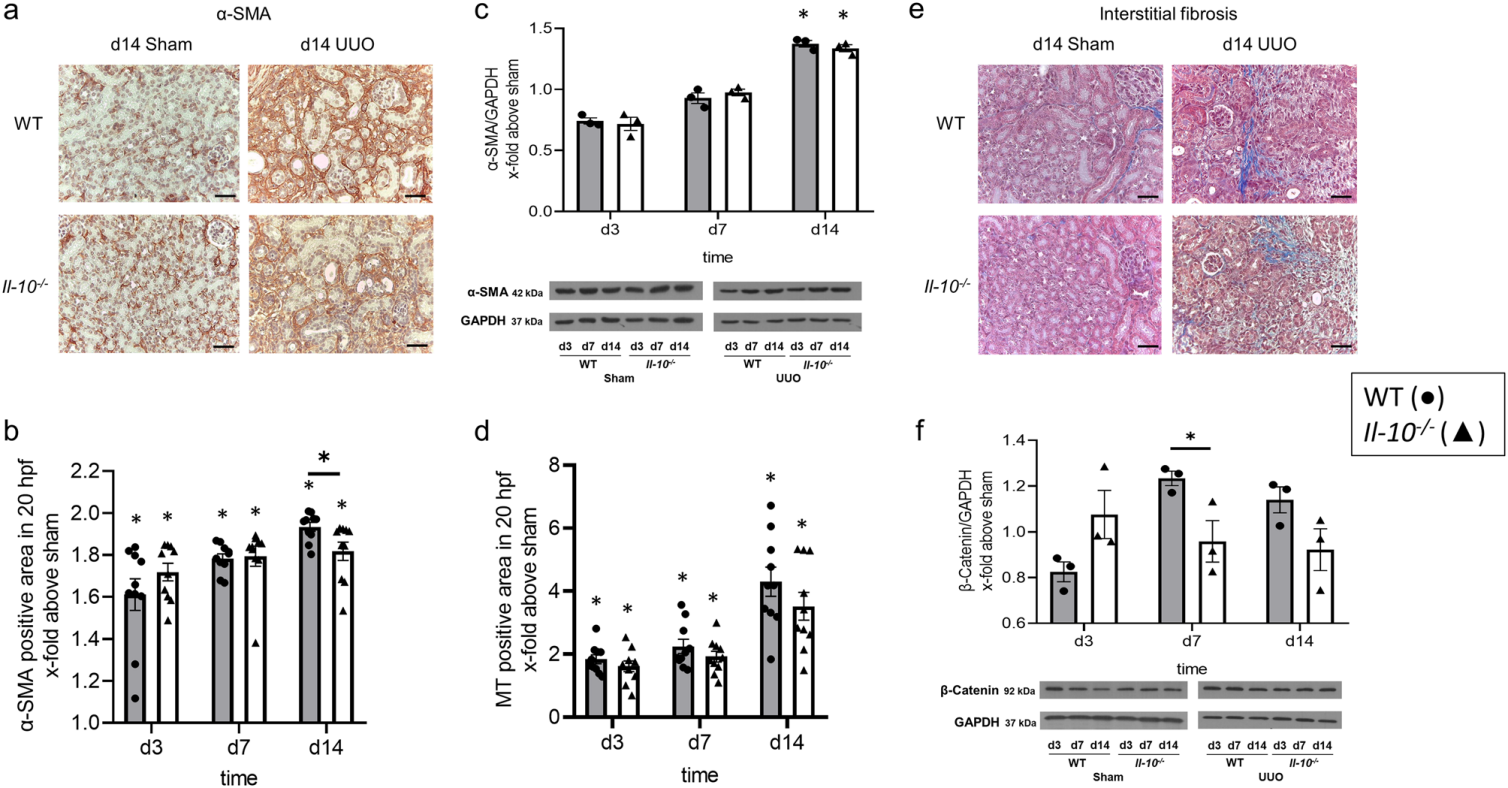
Interstitial fibrosis in neonatal UUO kidneys. Renal sections of UUO- and sham-operated neonatal kidneys were stained for α-SMA and Masson’s Trichrome (MT). UUO induced α-SMA expression in neonatal kidneys in WT and *Il-10*^*−/−*^ (**a**). α-SMA positive area was slightly reduced in *Il-10*^*−/−*^ on d14 (**b**). α-SMA protein expression, measured via western blot, also increased following UUO, but without significant differences between WT and *Il-10*^*−/−*^ (**c**). UUO induced collagen deposition in WT and *IL-10*^*−/−*^ kidneys (**d**,**e**). MT positive did not differ significantly between WT and *Il-10*^*−/−*^ (**d**,**e**). β-Catenin expression increased after UUO, *Il-10*^*−/−*^ showed less fibrosis on d7 than WT (**f**). The shown western blot images are cropped, for uncropped western blots see Supplementary Figs. S8, S9 online. Expression is indicated as x-fold increase above sham operated control; n = 3 for western blot analysis and n = 10 for immunohistochemical staining; *p < 0.05. Data are presented as individual points with mean ± SEM. Bar = 100 µm. Standalone * represents significant differences between Sham and UUO results.

To study interstitial fibrosis in *Il-10*^*−/−*^ and WT mice after neonatal UUO, α-SMA staining of kidney sections as well as its overall expression were measured. The abundance and expression of α-SMA increased continuously in neonatal *Il-10*^*−/−*^ and neonatal WT mice with obstruction. We observed a lower expression of α-SMA in neonatal *Il-10*^*−/−*^ kidneys on d14 in the immunohistochemical staining, however, this result could not be confirmed by western blot analysis. (Fig. 6a–c) (uncropped western blot image: Supplementary Fig. S11). Next, we assessed interstitial collagen deposition measured by the Masson’s Trichrome staining. Again, we found no significant differences between obstructed kidneys of neonatal *Il-10*^*−/−*^ and neonatal WT mice (Fig. 6d,e). Finally, we investigated UUO-induced β-catenin expression in the neonatal kidneys of *Il-10*^*−/−*^ and WT mice which was significantly different only at day 7 of life (Fig. 6f) (uncropped western blot image: Supplementary Fig. S12). Expression of these fibrosis markers in sham-operated kidneys only is displayed in Supplementary Fig. S13. From these findings we conclude that IL-10 does not play a major role in the development of renal fibrosis in neonatal kidneys with UUO.

## Discussion

Interleukin 10 is an anti-inflammatory and antifibrotic cytokine produced by a broad variety of cells^11,18^. Hence, we investigated a potential anti-inflammatory role of IL-10 in neonatal kidneys with obstructive nephropathy. Obstructive nephropathies belong to the congenital anomalies of kidneys and urinary tract (CAKUT) and are one of the leading causes for kidney failure in children^47,48^. Using *Il-10*^*−/−*^ mice, we demonstrate that IL-10 stimulates the recruitment of immune cells into the obstructed neonatal kidney. In addition, we show IL-10 dependent release of pro-inflammatory cytokines and chemokines within the obstructed neonatal kidney. This is in contrast to findings in adult mice with UUO where IL-10 was reported to exert anti-inflammatory effects in obstructed kidneys^23^. However, our study has its limitations, as the usage of *Il-10*^*−/−*^ and WT littermates was not possible due to the low age required for the UUO surgery. This may lead to a potential bias.

We also found that IL-10 is upregulated after UUO in serum samples of neonatal WT mice, which provides additional support of a potential role of this cytokine in modulating immune responses in obstructive nephropathy. Immune cells are important mediators of the inflammatory response after UUO^49–52^. Following obstruction, neonatal kidneys of *Il-10*^*−/−*^ mice displayed a reduced number of infiltrating leukocytes, especially neutrophils, CD11b^hi^ cells, F4/80^+^ cells, cDC1, and T-cells. By contrast, in adult mice with UUO the absence of IL-10 led to an increase in the number of infiltrated T-cells and F4/80^+^ cells in the obstructed kidneys^23^. This differential regulation in neonatal and adult mice suggests that IL-10 may have an influence on the overall development and/or recruitment of immune cells in the neonatal period, thus inducing a different immune response to UUO compared to adult mice. In *Il-10*^*−/−*^ mice cDC1 and CD11b^hi^ cells show a delayed infiltration into the obstructed kidney in comparison to WT, which emphasizes the possibility of a still unknown function of IL-10 in the development of the immune system during the neonatal period. In neonatal UUO, nephrogenesis is still ongoing with constant changes in gene regulation and composition of the immune system in the kidney^46,53^. Even under basal conditions several of our markers already show diminished expression in *Il-10*^*−/−*^ mice compared to WT.

IL-10 is known as an anti-inflammatory cytokine that limits innate immune responses mainly by inhibition of pro-inflammatory cytokines^11^. Since the markers we used to assess infiltration of leukocytes don’t assess their degree of activation, we also analyzed the chemokine and cytokine profiles in the neonatal kidneys of *Il-10*^*−/−*^ and WT mice following UUO. Surprisingly, neonatal *Il-10*^*−/−*^ mice with UUO showed a reduction in pro-inflammatory cytokine and chemokine content in obstructed kidneys when compared to WT with UUO, mainly of IP-10, IL-1α, MIP-2α,IL-17A, eotaxin, and ENA-78. These results are congruent with the reduction of infiltrating leukocytes into the neonatal kidney and demonstrate again the differential regulation of immune responses by IL-10 in the neonatal versus adult organism. Under inflammatory conditions a variety of cells can express the chemokines IP-10 and MIP-2α, which attracts inflammatory cells in different renal diseases^54–56^. Here, we show an increase in levels of IP-10 and MIP-2α following neonatal UUO, which was markedly reduced in neonatal *Il-10*^*−/−*^ mice in comparison to neonatal WT mice. Contrary to our findings, in the model of cisplatin nephrotoxicity IL-10 deficiency in adult mice has been shown to induce an increase of IP-10 levels in the kidney^24^. Additionally, IL-10 has been shown to decrease MIP-2α after infection^57,58^. In the adult UUO model, MIP-2α mRNA expression increased greatly in the obstructed kidney^59^. Low concentration of MIP-2α after neonatal obstruction may be also due to low concentration of IL-17A, an activator of MIP-2α production. IL17A induces cytokine production in renal epithelial cells^60^. Deficiency of IL-17A attenuated injury in a renal ischemia reperfusion model^61^. Following UUO in adult mice, IL-17A increased and induced renal fibrosis^62^. IL-10 has been shown to suppress IL-17A production in various models^58,63^. In the neonatal setting, we observed a significant reduction in IL-17A levels in obstructed kidneys of neonatal *Il-10*^*−/−*^ mice compared to neonatal WT mice. This could be related to the fact that IL-17A is mostly produced by T-cells which are reduced in number in obstructed neonatal kidneys of *Il-10*^*−/−*^ mice. We also investigated IL-1α, a cytokine which is a key mediator of sterile inflammation^4,64,65^. Neonatal UUO induced IL-1α release in WT, but not in *Il-10*^*−/−*^ kidneys. The observed increase of IL-1α in neonatal WT UUO kidneys is in line with our previous findings^4^. However, IL-10 has been shown to suppress IL-1α production by resident peritoneal macrophages in vitro^66^. This contrasts with our finding suggesting a differentially regulated interplay between IL-1α and IL-10 in neonatal and adult mice with UUO. The increased concentration of chemokines following UUO in WT in comparison to *Il-10*^*−/−*^ shows a possible mechanism driving the pro-inflammatory role of IL-10 in neonatal UUO. IP-10 is mostly known for its role in recruiting T cells^67^. MIP-2α, MIP-1β, and GM-CSF play a role in macrophage recruitment^68–70^. Eotaxin, although mostly associated with eosinophil recruitment, was shown to be critical in mammary gland development and also takes part in the inflammatory response in diabetic nephropathy^71,72^. Overall, the cytokines and chemokines we investigated here are also involved in neutrophil recruitment, especially ENA-78 being the epithelial neutrophil-activating protein^64,73–76^. The reduction of these chemokines in the obstructed kidneys of *Il-10*^*−/−*^ mice is in line with the observed reduction of the infiltration of neutrophils, macrophages, and T cells into the obstructed kidneys of neonatal *Il-10*^*−/−*^ mice. IL-10 promotes proliferation and activation of CD8^+^ T cells and thus release of cytokines and chemokines by these cells^16^. In a model of human endotoxemia an upregulation of interferon-γ and the chemokine IP-10 associated with it was observed after administration of recombinant human IL-10^77^. This may indicate that IL-10 is regulated differently in neonatal and adult mice with a pro-inflammatory function in the neonatal kidney via recruitment of immune cells through increased secretion of chemokines.

Our findings propose a differential regulation of IL-10 in the neonatal period. In fact, the neonatal immune system differs significantly from the adult one with marked suppression of pro-inflammatory canonical NF-κB signaling and activation of anti-inflammatory non canonical NF-κB signaling, as recently demonstrated^77^. This shifted balance reflects a fine-tuned adaption during the transition from fetal life in a protected environment to postnatal life with the sudden exposition of the neonate to a microbial-rich outside world^78^. Interestingly, in a sepsis model it has been shown that infants have a diminished response to IL-10 and the expression of the IL-10 receptor is strongly reduced in neonatal T-cells^79,80^. Recently it has been shown that IL-4 enhances IL-10 production, and the lack of its receptor inhibits IL-10 production in Th1 cells^81^. The concentration of IL-4 we measured in neonatal obstructed kidneys did show neither a response to UUO nor a difference between *Il-10*^*−/−*^ and WT. IL-4 is also a product of the IL-10 pathway, the fact that it’s not upregulated after UUO in our model indicates that the mechanism of IL-10 production and its effects in response to injury are differentially regulated in neonates and adults. Future studies are warranted to uncover the molecular mechanisms of IL-10 driven pro-inflammatory response to injury in neonatal development.

Cell death is an important hallmark of UUO. UUO induces apoptosis in the developing kidney with obstruction. However, we did not observe a difference in full length PARP expression following UUO in both neonatal *Il-10*^*−/−*^ and WT kidneys indicating that IL-10 has no impact on the induction of apoptosis in neonatal UUO. TUNEL staining of tubular apoptotic nuclei further confirmed our findings regarding apoptosis. Recently it has been shown that ER stress induced by UUO in adult mice is a major source for apoptosis in this model and IL-10 protects the kidney by suppressing ER stress in adult mice^42^. In our study we show that neonatal UUO induces ER stress in the kidney through GRP78/BiP expression and we also observed for one time point (day 7) more ER stress in *Il-10*^*−/−*^ in comparison to WT. However, for the other time points we found no differences suggesting that IL-10 has no impact on ER stress in neonatal UUO. Apoptosis is highly upregulated during nephrogenesis^82^, given this slight differences between the transgenic lines may not be detectable in our model.

Besides apoptosis we also measured necroptosis, a pro-inflammatory form of regulated necrosis, in which RIPK3 is involved^83,84^. Following UUO, the protein expression of RIPK3 increased in neonatal WT kidneys. However, *Il-10*^*−/−*^ did not show this increase which then also became significant at day 14. Whether TGF-β is involved here is not clear at the moment. TGF-β has been described to activate RIPK3-dependent cell death pathways leading to necroptosis^85^. Our results did not find a significant difference, but a decreasing trend in TGF-β expression in obstructed kidneys of *Il-10*^*−/−*^ versus WT mice.

End-stage outcome of the UUO model is severe interstitial fibrosis in the obstructed kidney. The basis for fibrotic diseases consists of expansion of connective tissue and abnormal deposition of fibrotic collagen fibers. The main source of extracellular matrix in renal fibrosis are myofibroblasts^86^. We measured the quantity of fibrotic collagen fibers, as well as evaluated myofibroblasts in neonatal *Il-10*^*−/−*^ and WT kidneys. The results show an overall increase in fibrotic fibers and α-SMA expression (a myofibroblast marker after UUO) with no significant differences between *Il-10*^*−/−*^ and WT mice. β-catenin, which is highly involved in nephrogenesis and kidney fibrosis, was reduced at one point in *Il-10*^*−/−*^ mice, suggesting an amelioration, however the significant difference vanished over time^87^. Deletion of IL-10 in adult UUO mice had promoted α-SMA accumulation, as well as collagen deposition in the adult mouse kidney after obstruction^42^. Overall, we did not find considerable differences in renal fibrosis development between obstructed kidneys in neonatal *Il-10*^*−/−*^ versus WT mice suggesting that IL-10 does not play a major role in modulating renal fibrosis in neonatal obstruction, which is contrasting the functional role of IL-10 in adult mice with UUO. Renal fibrosis following ureteral obstruction is more severe in neonatal compared to adult mice^6,88,89^. The amount of extracellular matrix deposition in the neonatal kidneys may be too strong to detect slight differences coming from a differentially regulated immune response by the loss of IL-10. In addition, the effect of the IL-10 deletion with less immune cell infiltration in the kidney could be only transient and overall too weak to reduce interstitial fibrosis in neonatal UUO.

## Conclusion

We show that IL-10 plays a critical role in the recruitment of immune cells and concomitant cytokine release in obstructed neonatal kidneys. However, and in contrast to adult mice with obstruction, deficiency of IL-10 seems to have an anti-inflammatory and recruitment inhibitory effect in neonatal kidneys after obstruction accompanied with diminished release of pro-inflammatory cytokines. Notably, IL-10 does not have a substantial effect on cell death and interstitial fibrosis in the neonatal UUO model highlighting the differential and in part opposing role IL-10 plays in obstructed kidneys of neonatal and adult mice. Further investigations are now warranted to clarify the functional role of IL-10 and the mechanism behind its pro-inflammatory function in neonatal versus adult UUO and beyond.

### Supplementary Information

Supplementary Figures.

## Data Availability

The datasets generated during and/or analyzed during the current study are available from the corresponding author on reasonable request.
