# Supplementary material for: Interleukin-10 enhances recruitment of immune cells in the neonatal mouse model of obstructive nephropathy
Source: Sci Rep. 2024 Mar 6;14:5495. doi: 10.1038/s41598-024-55469-9 (PMC10917785; doi:10.1038/s41598-024-55469-9)
Supplement: Supplementary file 1 — Supplementary Figures. [file 41598_2024_55469_MOESM1_ESM.pdf]

Supplementary Material

Interleukin-10 enhances recruitment of immune cells in the neonatal mouse model of obstructive nephropathy

Maja Wyczanska[1], Franziska Thalmeier[1], Ursula Keller[1], Richard Klaus[1], Hamsa Narasimhan[2], Xingqi Ji[2], Barbara U. Schraml[2], Lou M. Wackerbarth[3], Bärbel Lange-Sperandio[1*]

[1]Department of Pediatrics, Dr. v. Hauner Children's Hospital, University Hospital, LMU Munich, 80337 Munich, Germany

[2]Walter-Brendel-Centre of Experimental Medicine, University Hospital, LMU Munich, 82152 Planegg-Martinsried, Germany

[3]Biomedical Center, Institute for Cardiovascular Physiology and Pathophysiology, Faculty of Medicine, LMU Munich, 82152 Planegg-Martinsried, Germany

***Correspondence**:
Bärbel Lange-Sperandio, M.D.
Dr. v. Hauner Children's Hospital, LMU Munich
Lindwurmstraße 4
80337 Munich, Germany
Phone: +49 (0)89/4400 5 2811
Fax: +49 (0)89/4400 5 2771
Email: baerbel.lange-sperandio@med.uni-muenchen.de

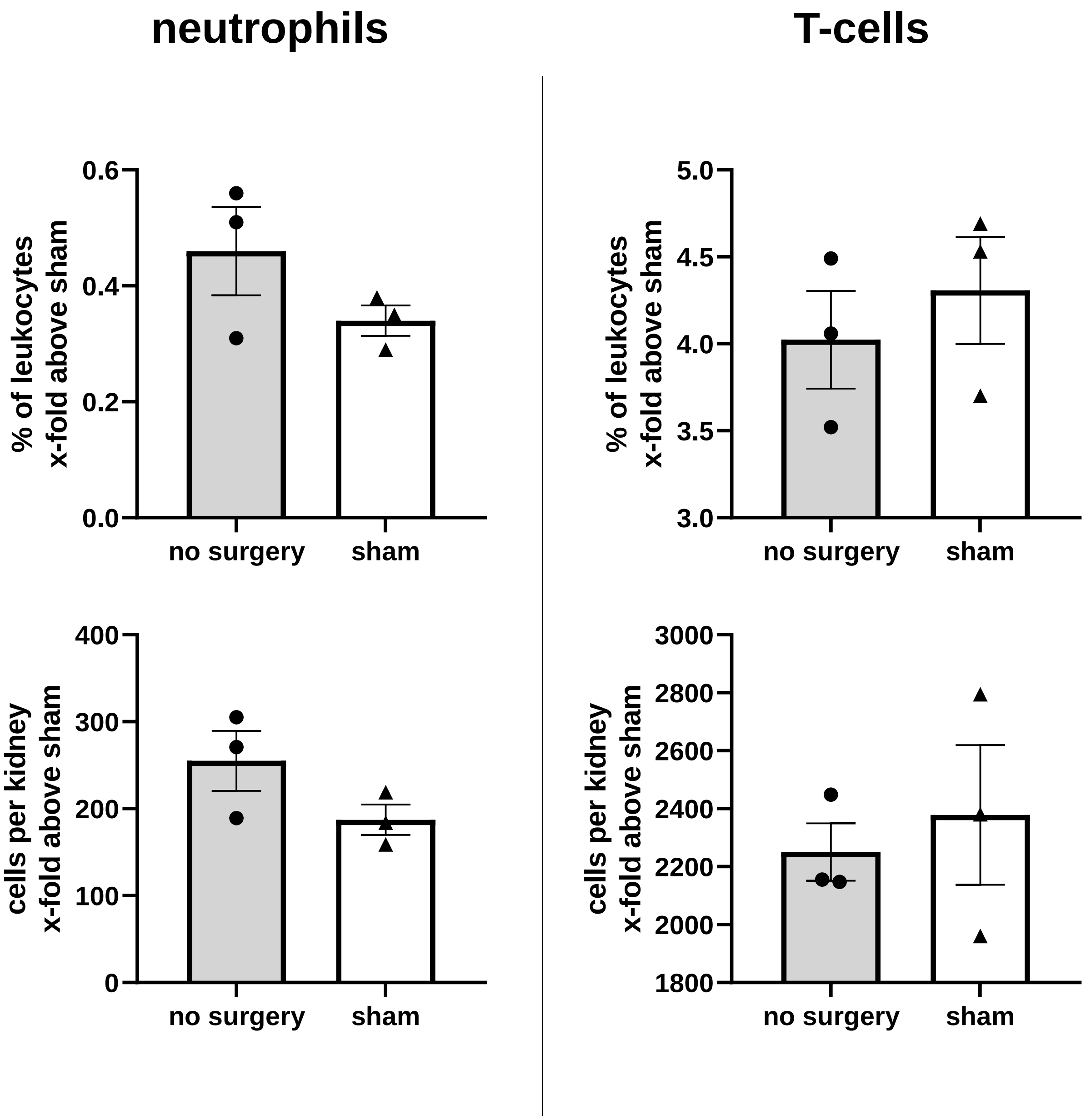

Supplementary figure S1

FACS steady state analysis for neutrophils and T-cells in non-operated (light gray) and sham-operated (white) WT kidneys. There are no significant differences in the neutrophils (a) and T-cells (b) frequency and cell number between non-operated and sham-operated kidneys. n=3; *p<0,05. Data are presented as mean +/- SEM.

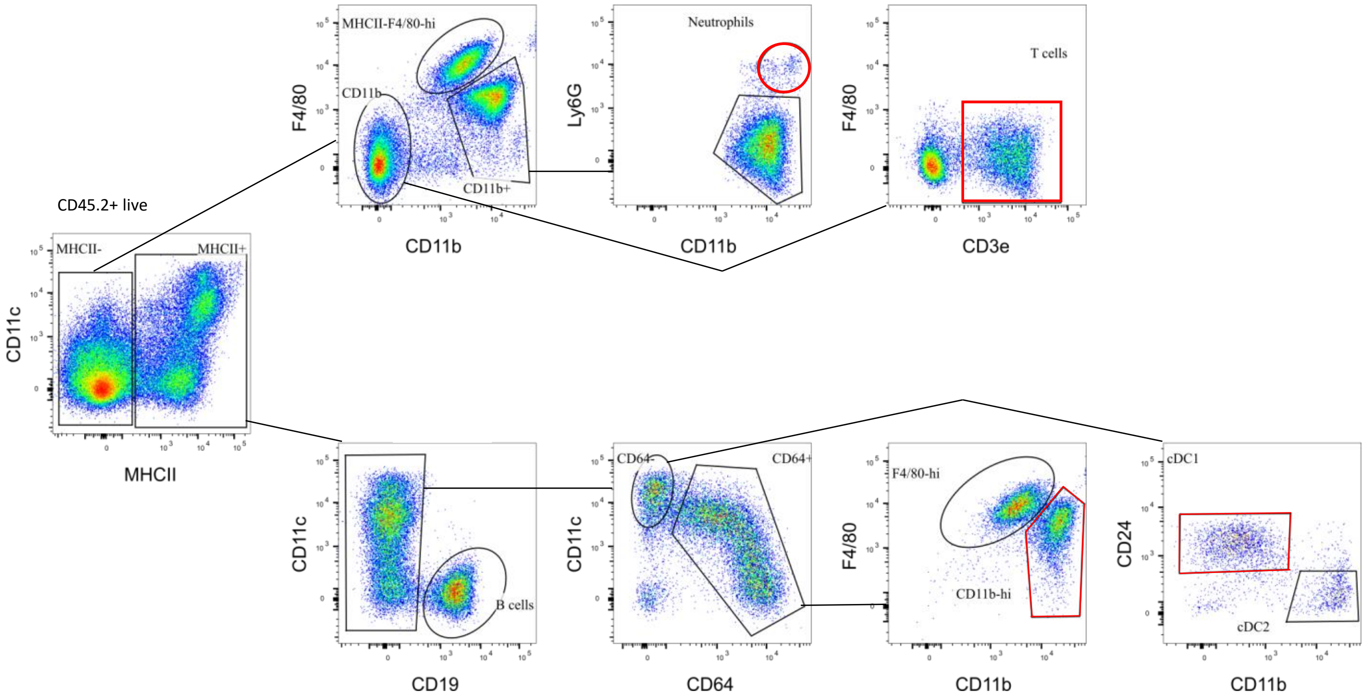

Supplementary figure S2

Gating strategy for neutrophils, macrophages, dendritic cells and T-cells. Live CD45.2^{+} MHCII^{-} and MHCII^{+} cells were gated as indicated and MHCII^{-} cells were analyzed for F4/80 and CD11b expression and subdivided in CD11b^{+} and CD11b^{-} cells. CD11b^{+} cells were further analyzed for Ly6G and CD11b expression to identify neutrophils. CD11b^{-} cells were further analyzed for F4/80 and CD3 expression to identify T-cells. MHCII^{+} cells, excluding Ly6c^{+} cells, were analyzed for CD11c and CD19 expression to identify non-B-cells, of which expression of CD11c and CD64 was analyzed to identify CD11b^{hi} cells through expression of F4/80 and CD11b. Out of CD64- cells the expression of CD24 and CD11c was used to identify cDC1. Data from sham-operated controls was used to visualize the gating strategy.

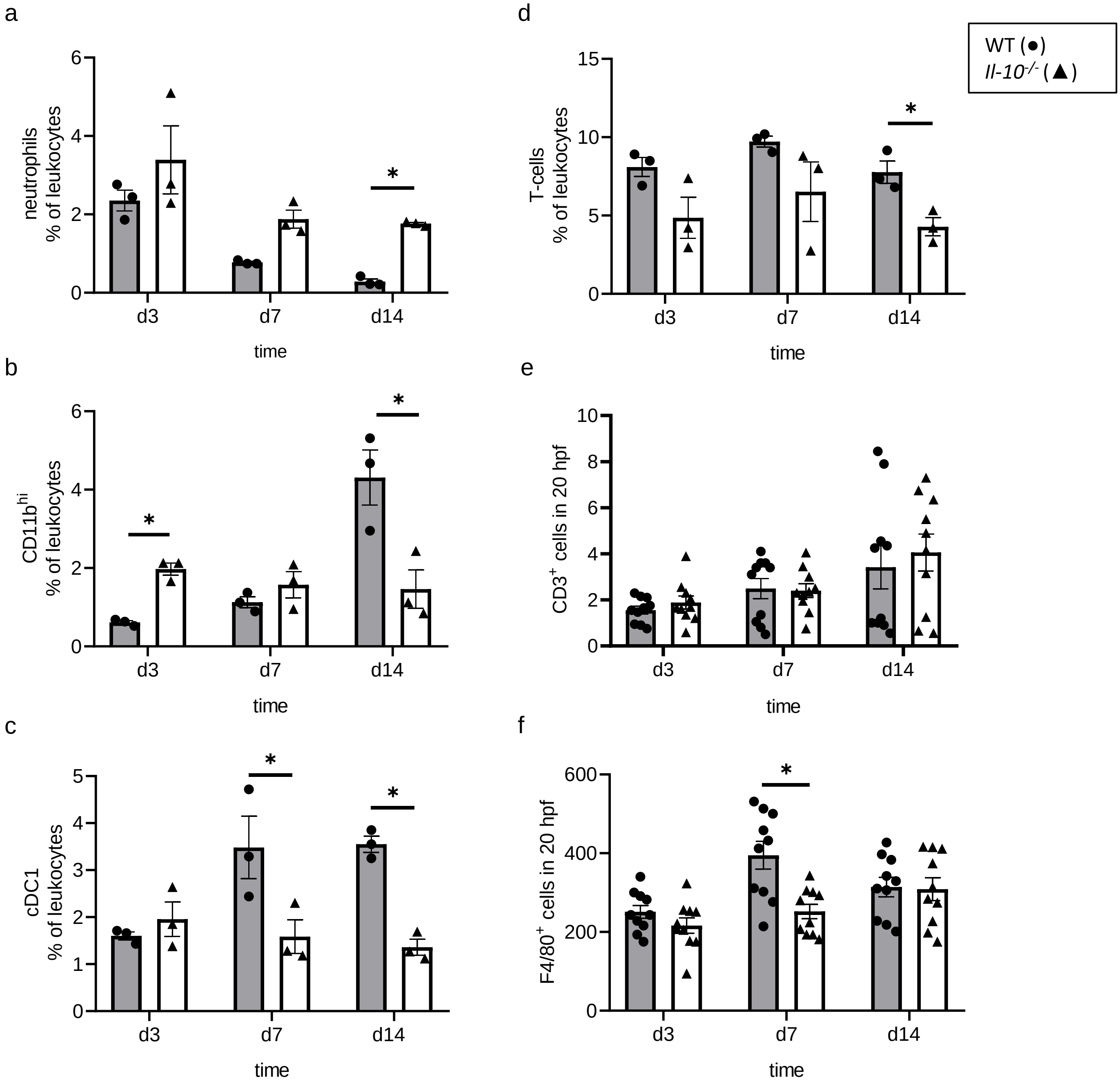

Supplementary figure S3

Analysis of neutrophiles, macrophages, dendritic cells and T-cells in sham-operated kidneys. Neonatal mice were subjected to sham operation. Frequency of renal Ly6G$^+$ neutrophils (a), CD11b^{hi} cells (b), cDC1 dendritic cells (c), and CD3$^+$ T-cells (d) in sham-operated kidneys at the indicated ages are shown. Neutrophils frequency is significantly higher in *Il-10*$^{-/-}$ than in WT at d14 (a). Frequency of CD11b^{hi} cells is higher in *Il-10*$^{-/-}$ than WT at d3, but lower at d14 (b). Frequency of dendritic cells at d7 and d14 (c), and of T-cells at d14 (d) is lower in *Il-10*$^{-/-}$ compared with WT. Additionally immunohistological staining results for CD3 (T-cells) and F4/80 (macrophages and dendritic cells) of WT and *Il-10*$^{-/-}$ sham mice are shown (e and f). CD3$^+$ cells (e), as well as F4/80$^+$ cells (f) did not show significant differences between *Il-10*$^{-/-}$ and WT in sham-operated neonatal mouse kidneys. n=3 (a-d), n=10 (e and f); *p<0,05. Data are presented as individual points with mean +/- SEM.

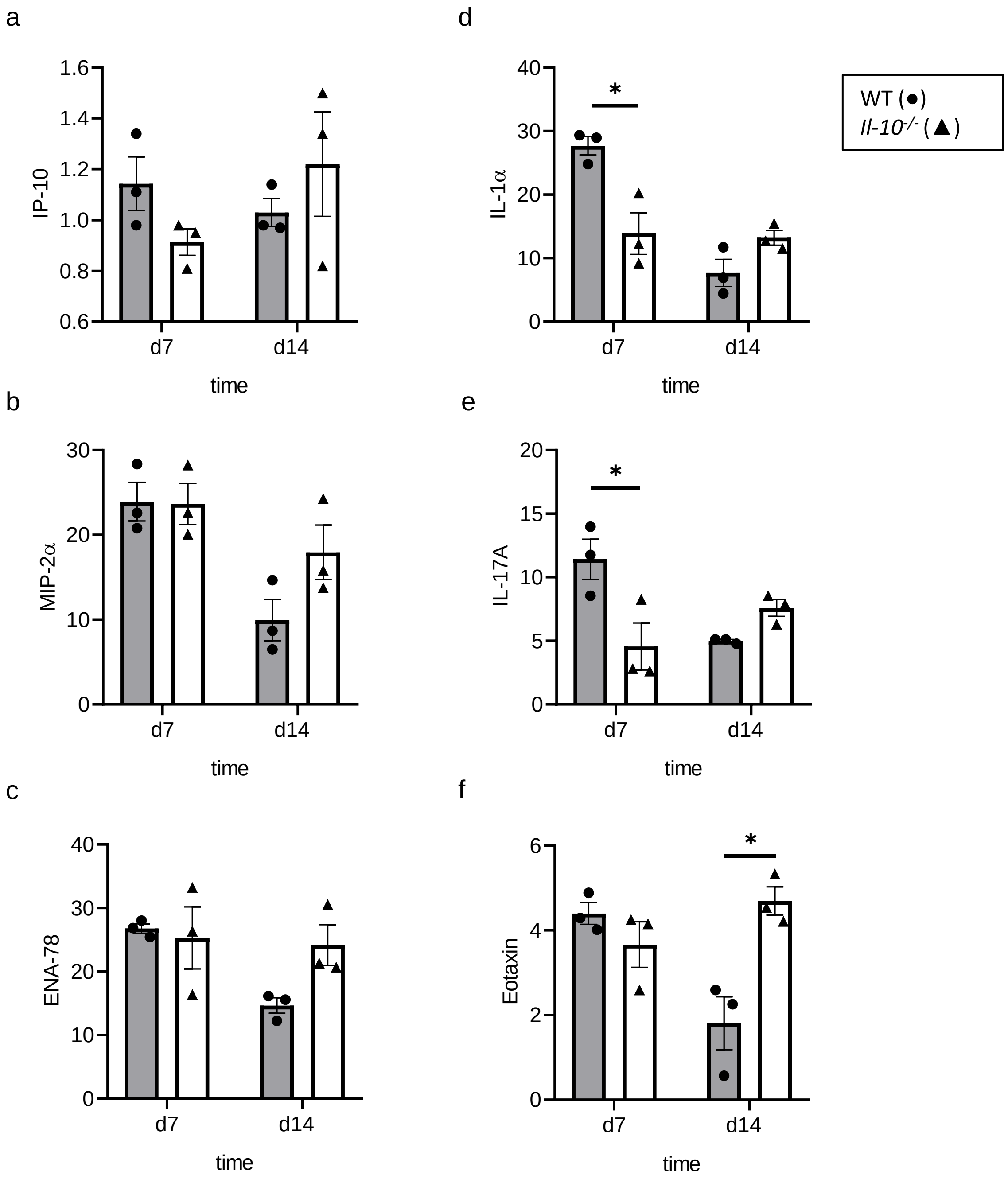

Supplementary figure S4

Cytokine and chemokine concentration in sham-operated neonatal kidneys. Whole sham-operated of neonatal mice were harvested and analyzed for cytokine and chemokine concentration. IP-10/CXCL10 (a), MIP-2α/CXCL2 (b) and ENA-78 (c) concentration in sham-operated kidneys was not different between *Il-10*$^{-/-}$ and WT. IL-1α (d) and IL-17A (e) concentration was lower in *Il-10*$^{-/-}$ kidneys in comparison to WT at d7 but without significant differences at d14. Eotaxin/CCL11 concentration decreased over time in WT mice, but not in *Il-10*$^{-/-}$ mice (f). n=3; *p<0,05. Data are presented as individual points with mean +/- SEM.

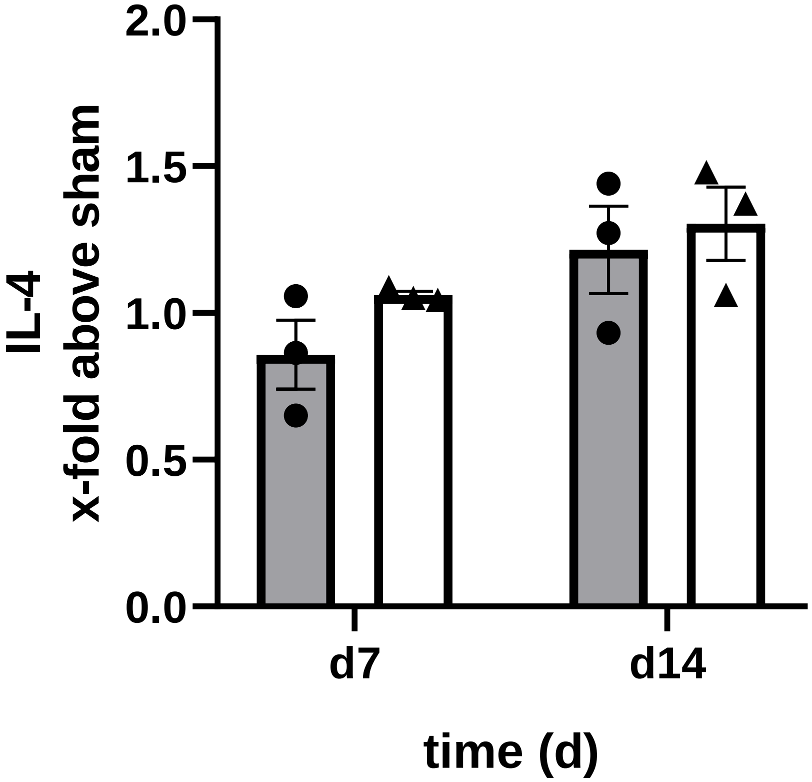

Supplementary figure S5

Concentration of Interleukin-4 in IL-10$^{-/-}$ and WT kidneys after UUO. There are no significant differences in the IL-4 concentration between IL-10$^{-/-}$ and WT kidneys after UUO on day 7 and 14 of life. Concentration is indicated as x-fold increase above sham-operated control; n=3; *p<0,05. Data are presented as mean +/- SEM.

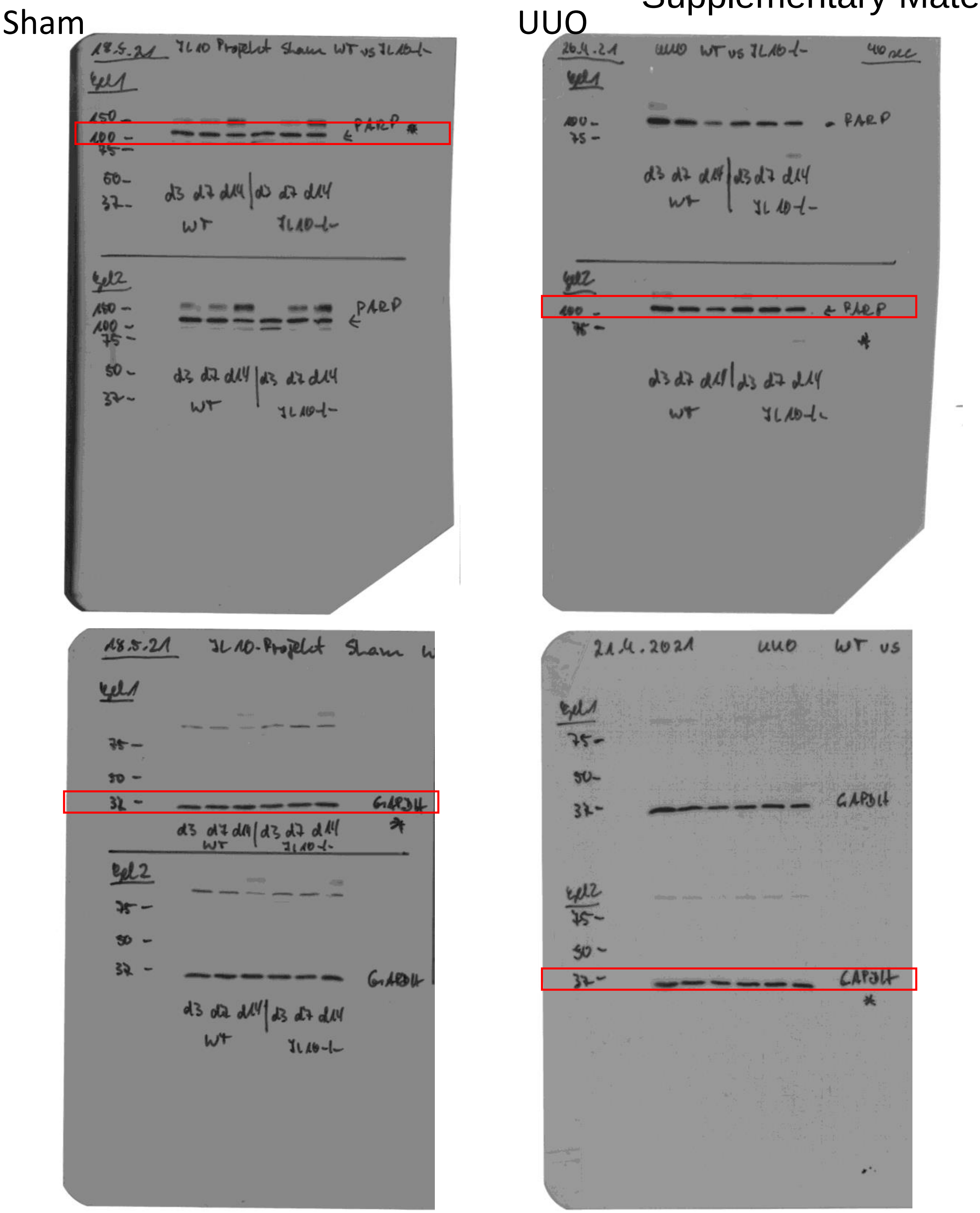

Supplementary figure S6

Western blot images for PARP and GAPDH in Sham (left) and UUO (right) kidneys. Uncropped western blot gel images for PARP and GAPDH in neonatal WT and IL-10$^{-/-}$ kidneys (on day 3, 7 and 14 of life). * and red box mark the section used in Figure 6. PARP and GAPDH were visualized separately, but they represent the same gel.

Sham UUO

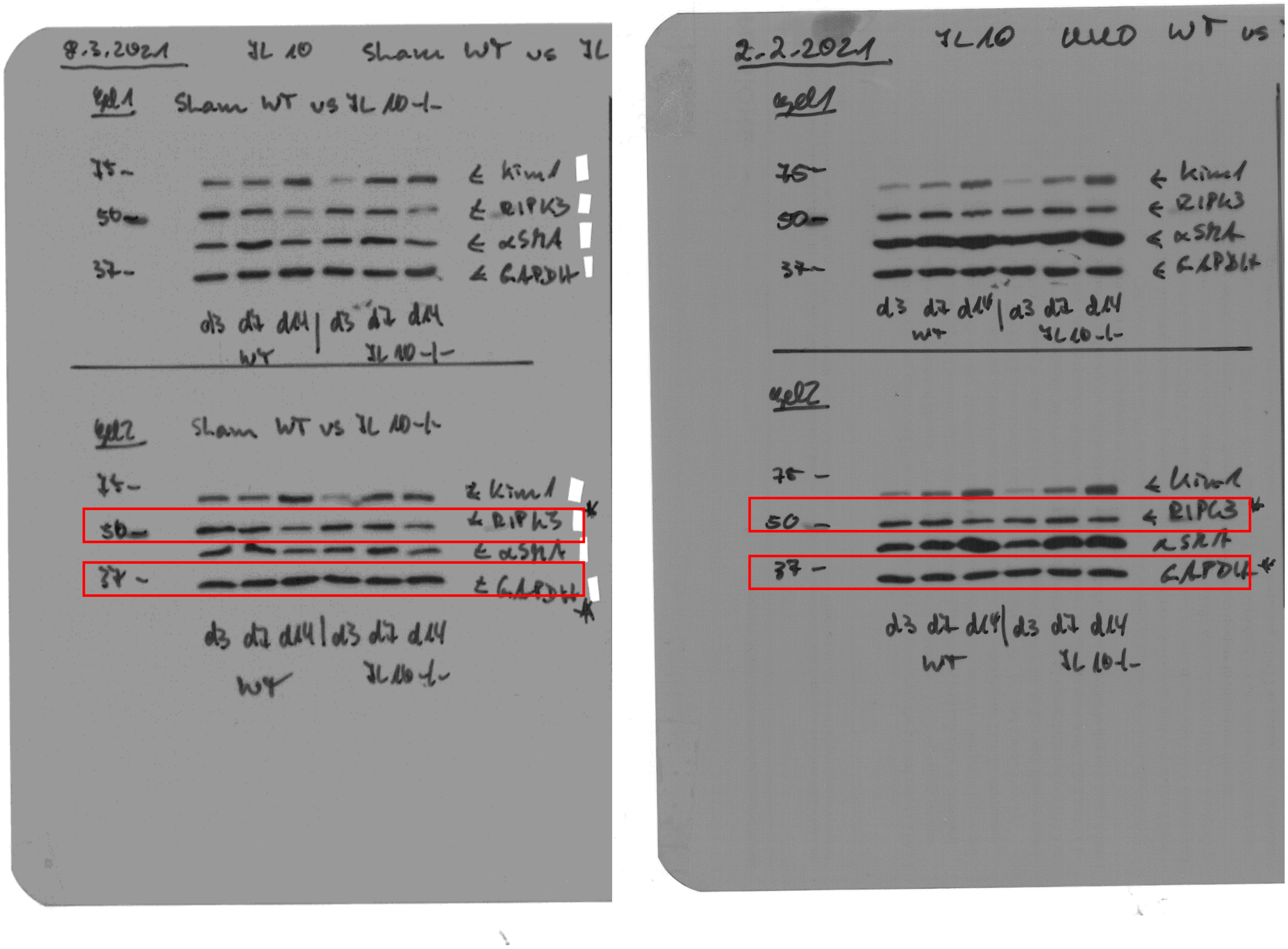

Supplementary figure S7

Western blot images for RIPK3 and GAPDH in Sham (left) and UUO (right) kidneys. Uncropped western blot gel images for RIPK3 and GAPDH in neonatal WT and IL-10$^{-/-}$ kidneys (on day 3, 7 and 14 of life). * and red box mark the section used in Figure 6.

Sham

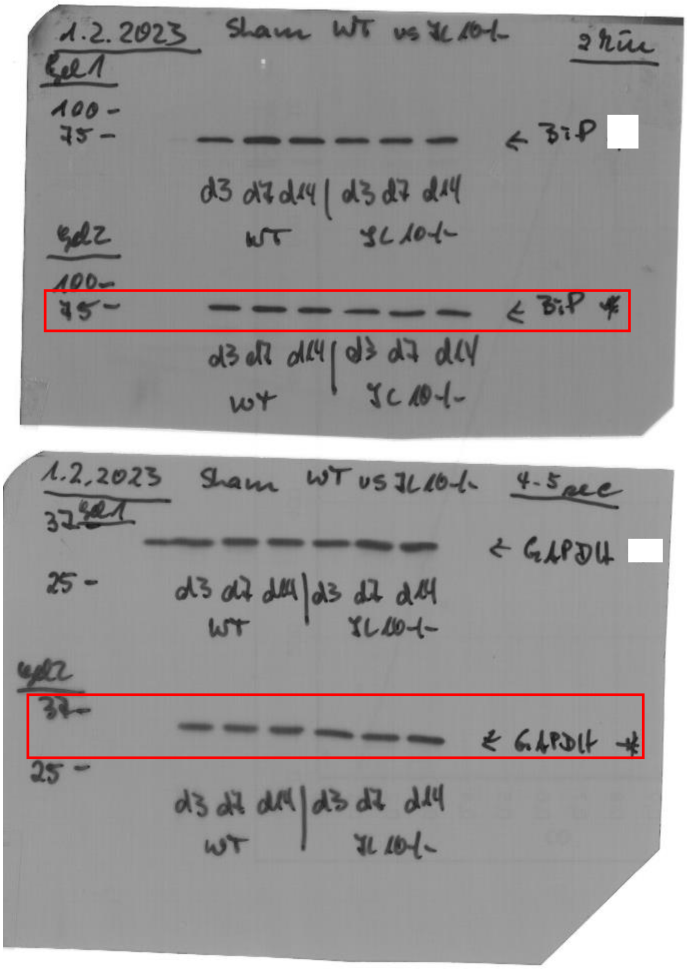

UUO

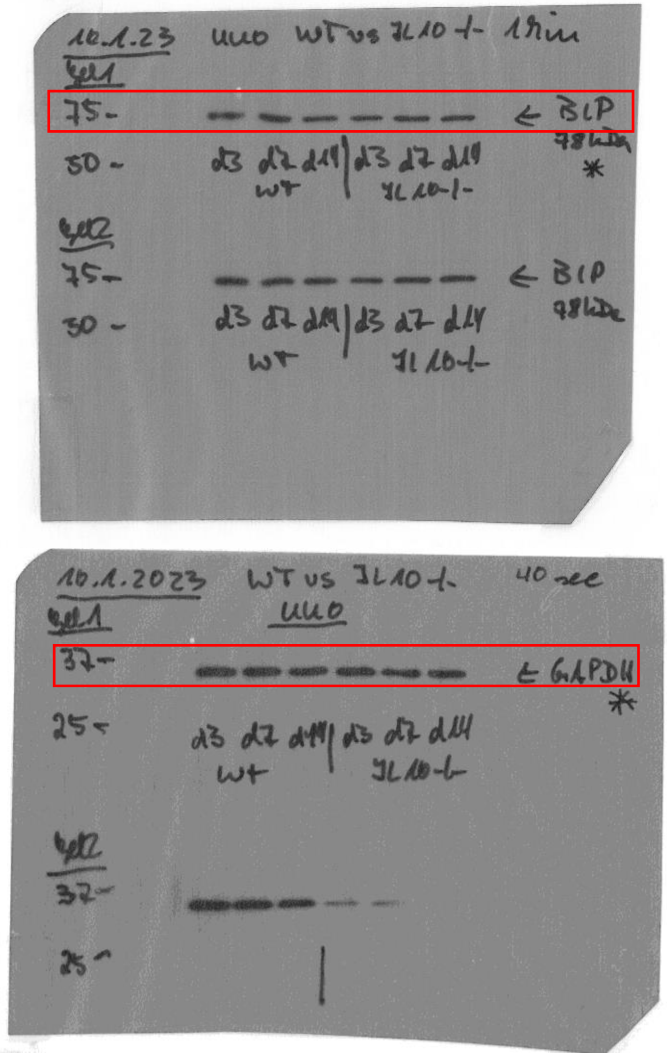

Supplementary figure S8

Western blot images for GRP78/BiP and GAPDH in Sham (left) and UUO (right) kidneys. Uncropped western blot gel images for GRP78/BiP and GAPDH in neonatal WT and IL-10$^{-/-}$ kidneys (on day 3, 7 and 14 of life). * and red box mark the section used in Figure 6. GRP78/BiP and GAPDH were visualized separately, but they represent the same gel.

Sham UUO

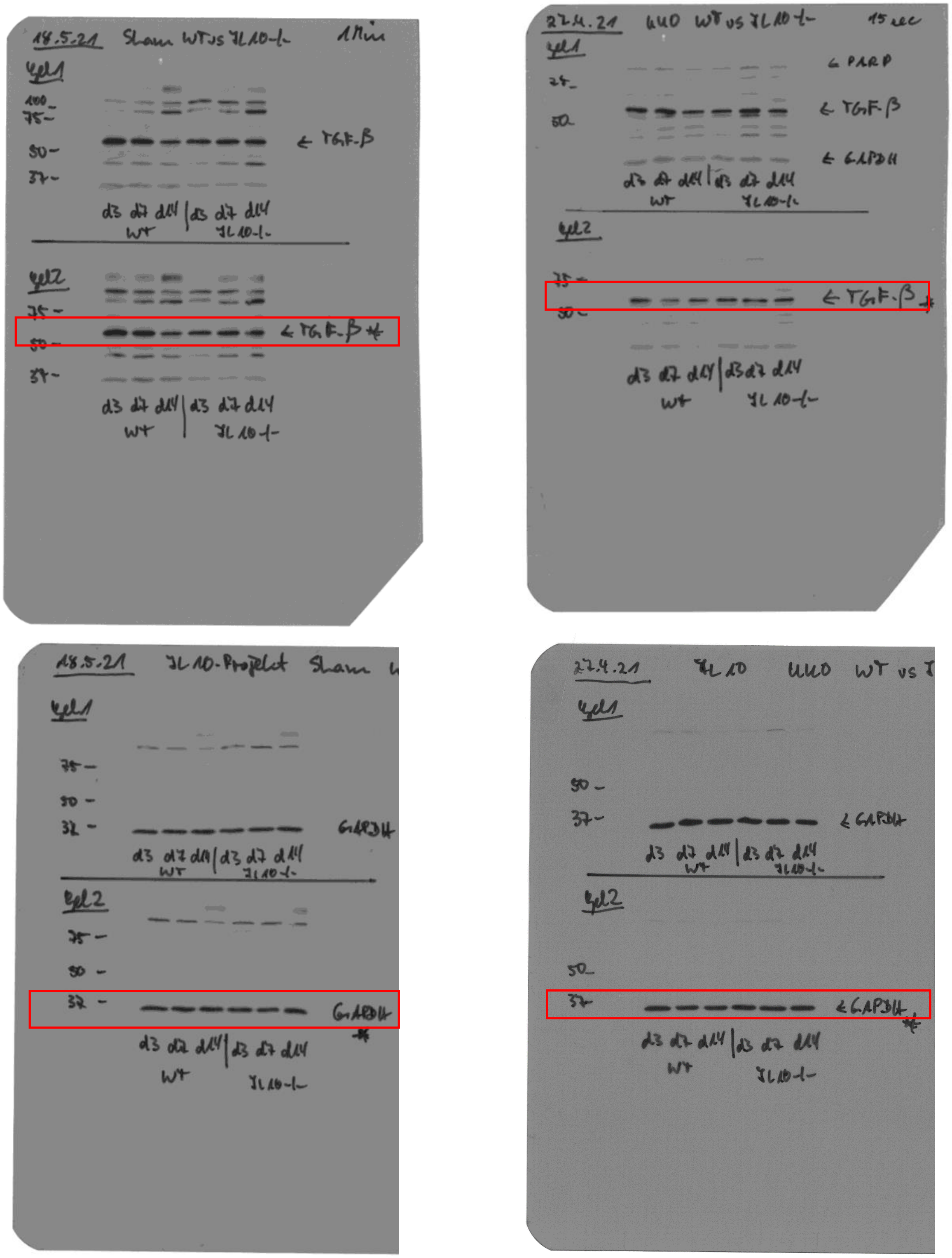

Supplementary figure S9

Western blot images for TGF-β and GAPDH in Sham (left) and UUO (right) kidneys. Uncropped western blot gel images for TGF-β and GAPDH in neonatal WT and IL-10$^{-/-}$ kidneys (on day 3, 7 and 14 of life). * and red box mark the section used in Figure 6. TGF-β and GAPDH were visualized separately, but they represent the same gel.

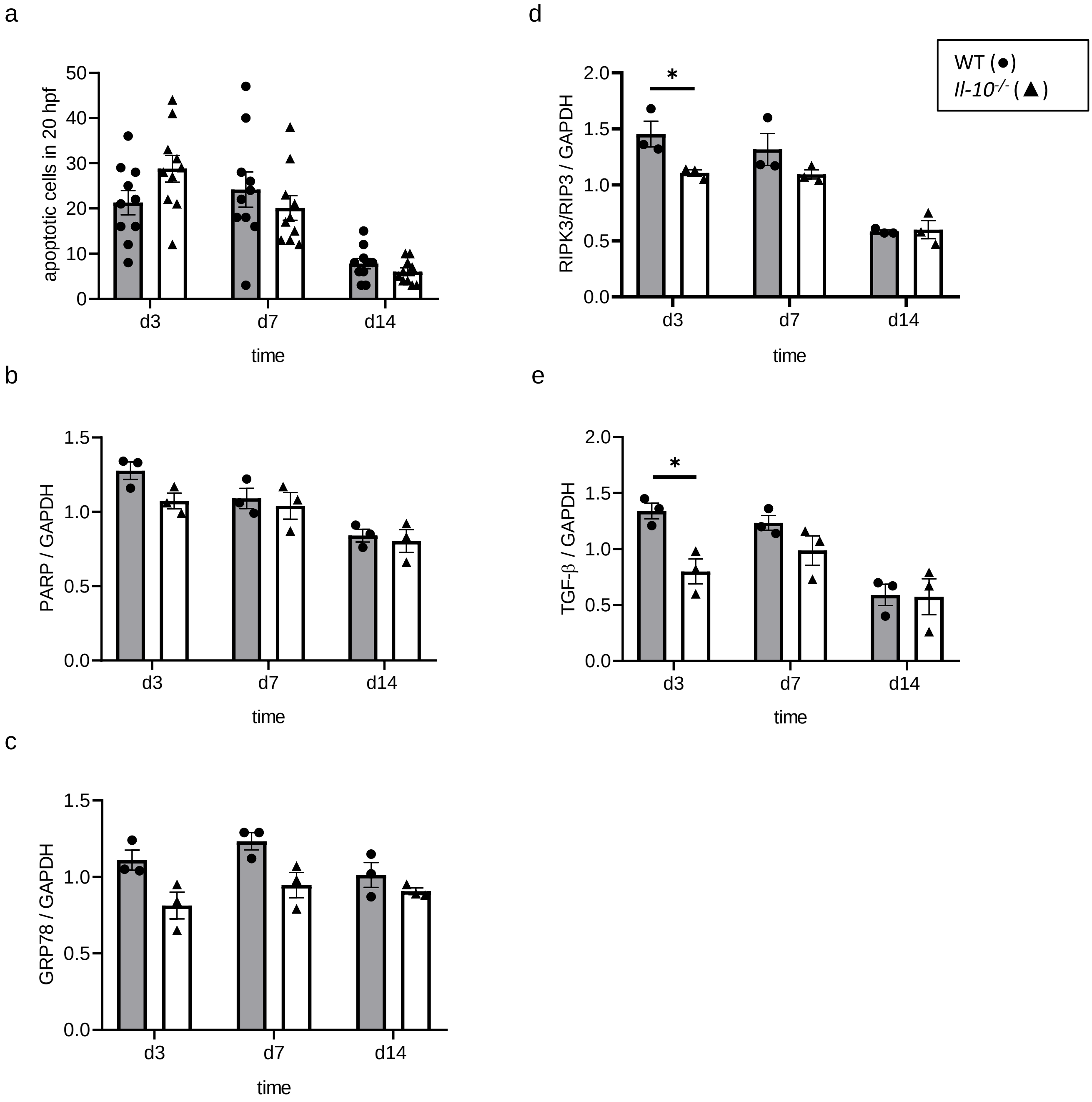

Supplementary figure S10

Cell death in neonatal *Il-10$^{-/-}$* sham-operated kidneys in comparison to WT. Apoptotic cells in sham-operated and UUO kidneys were detected by TUNEL staining in sections. Number of tubular apoptotic nuclei did not differ between *Il-10$^{-/-}$* and WT sham-operated kidneys (b). Whole kidneys were processed for western blot analysis at day 3, 7 and 14. Expression of PARP (b) and GRP78/BiP (c) were not significantly different between *Il-10$^{-/-}$* and WT mice. Expression of RIPK3 (d) and TGF-β (e) were lower in *Il-10$^{-/-}$* mice compared to WT after sham-operation on d3 but did not show differences at later time points. n=3 for western blot analysis and n=10 for immunohistochemical staining; *p<0,05. Data are presented as individual points with mean +/- SEM.

Sham

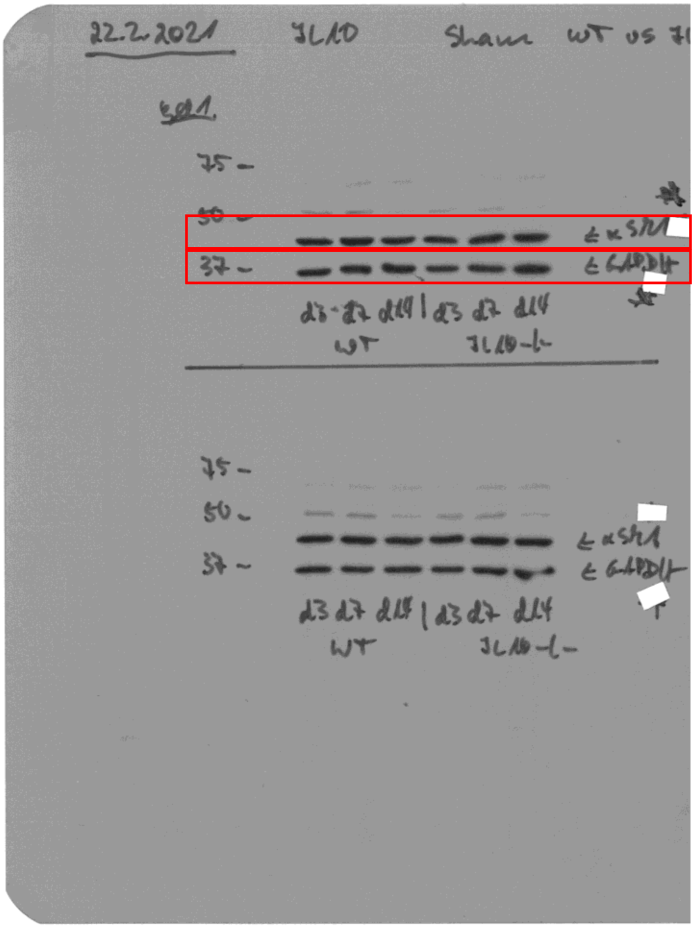

UUO

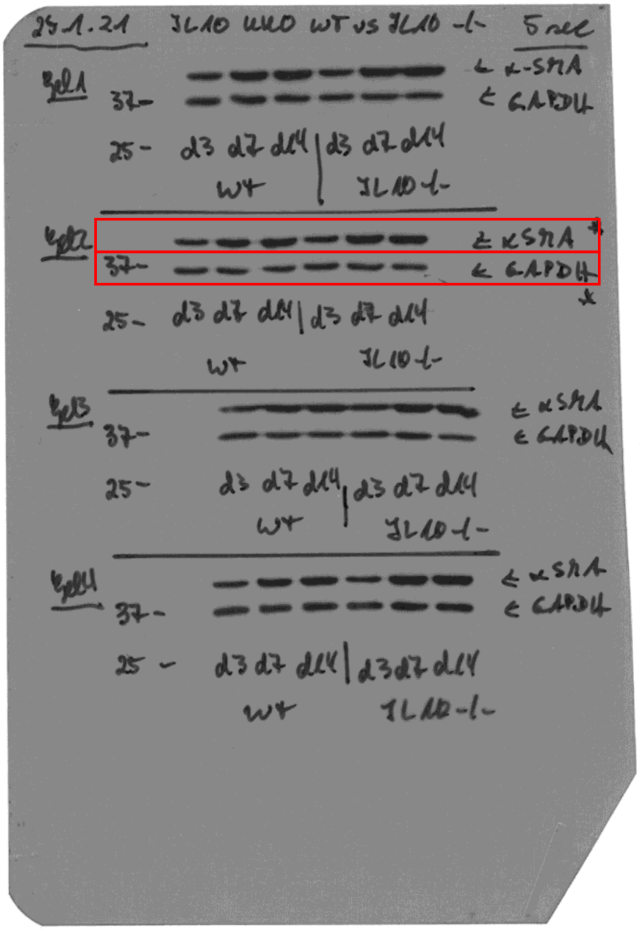

Supplementary figure S11

Western blot images for α-SMA and GAPDH in Sham (left) and UUO (right) kidneys. Uncropped western blot gel images for α-SMA and GAPDH in neonatal WT and IL-10$^{-/-}$ kidneys (on day 3, 7 and 14 of life). * marks the section used in Figure 7.

Sham UUO

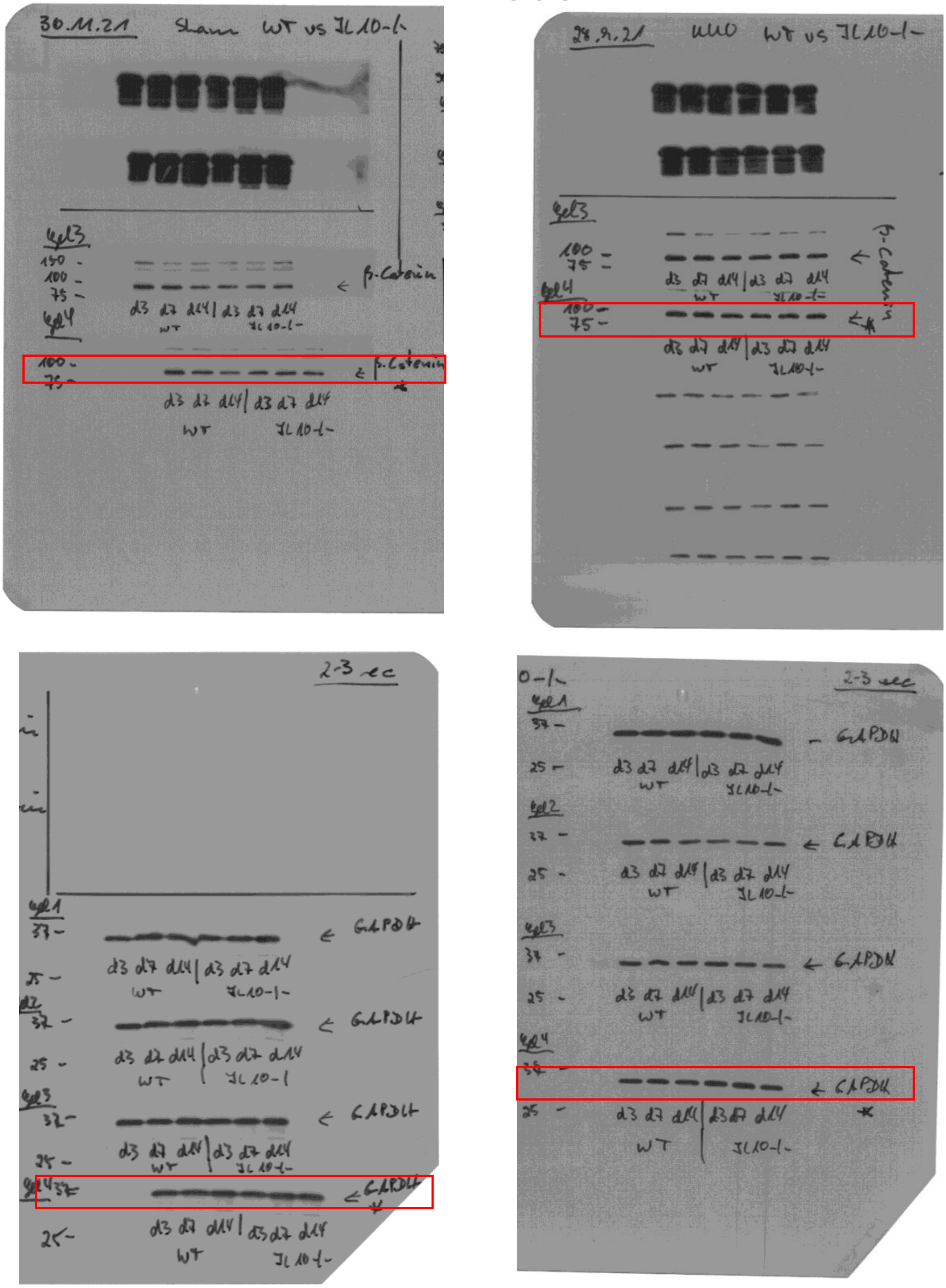

Supplementary figure S12

Western blot images for β-Catenin and GAPDH in Sham (left) and UUO (right) kidneys. Uncropped western blot gel images for β-Catenin and GAPDH in neonatal WT and IL-10$^{-/-}$ kidneys (on day 3, 7 and 14 of life). * marks the section used in Figure 7. β-Catenin and GAPDH were visualized separately, but they represent the same gel.

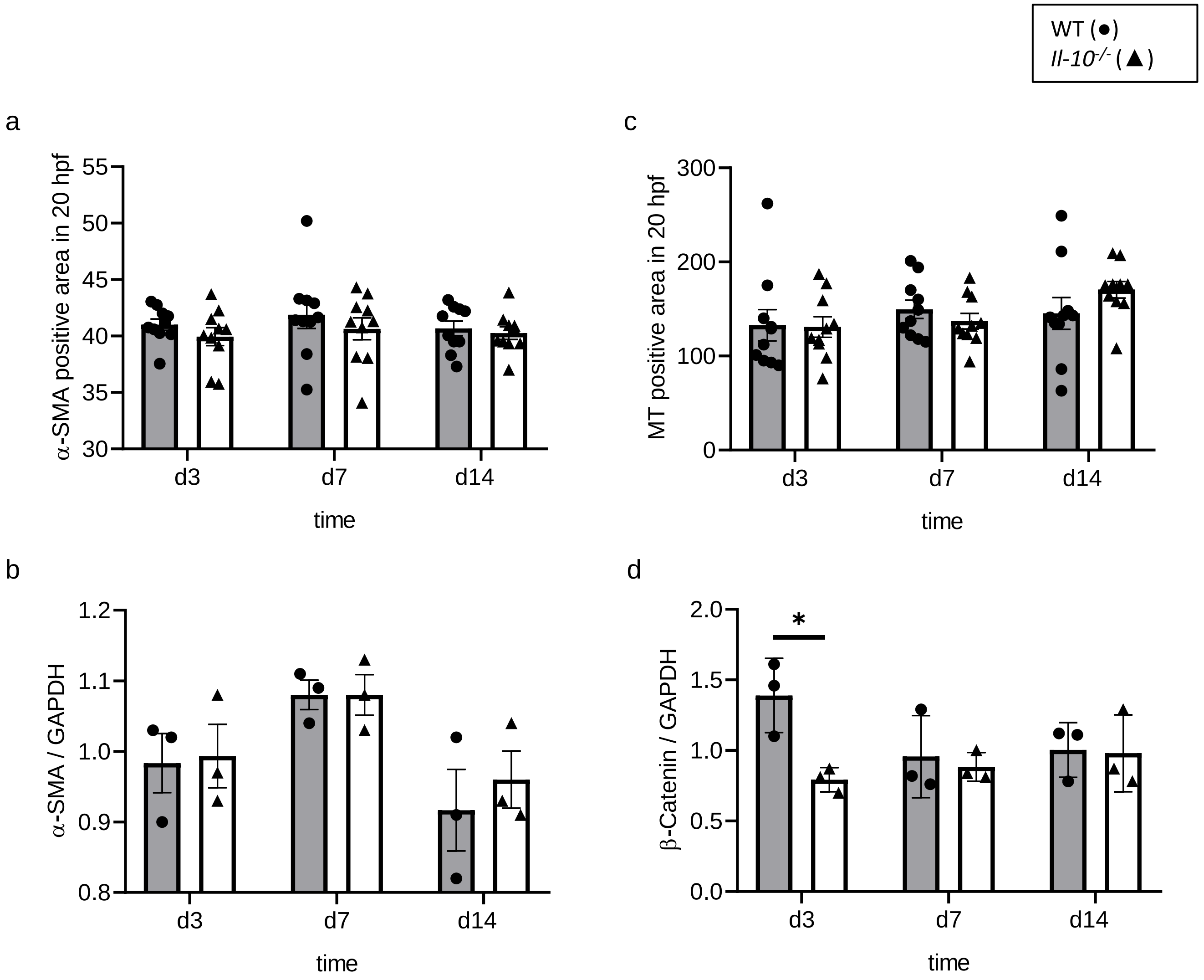

Supplementary figure S13

Interstitial fibrosis in neonatal sham-operated kidneys. Renal sections of sham-operated neonatal kidneys were stained for α-SMA and Masson's Trichrome (MT). α-SMA expression did not differ between *IL-10*$^{-/-}$ and WT kidneys (a). Neither did α-SMA (b) nor MT (c) positive area. β-Catenin expression was lower in *IL-10*$^{-/-}$ in comparison to WT mice at d3 but did not differ at later time points (d). n=3 for western blot analysis and n=10 for immunohistochemical staining; *p<0,05. Data are presented as individual points with mean +/- SEM.
